# Interplay of host and viral genetic variations in modulating antibody responses to genotype 3a hepatitis C virus: Implications for vaccine design

**DOI:** 10.1016/j.celrep.2025.116418

**Published:** 2025-10-10

**Authors:** Zhiqing Wang, Isla Humphreys, Jocelyn Quistrebert, Haiting Chai, Robert Stass, Josh Dhir, Alexandru Nisioi, Paul Radford, STOP-HCV consortium, Jonathan K. Ball, William L. Irving, Thomas A. Bowden, Paul Klenerman, Eleanor Barnes, Jane A. McKeating, Alexander W. Tarr, M. Azim Ansari

**Affiliations:** 1 Peter Medawar Building for Pathogen Research, University of Oxford, Oxford OX1 3SY, UK; 2 Chinese Academy of Medical Science Oxford Institute, University of Oxford, Oxford OX3 7BN, UK; 3 Institute of Infection and Immunity, The University of Birmingham, Birmingham B15 2TT, UK; 4 Division of Structural Biology, Centre for Human Genetics, University of Oxford, Oxford OX3 7BN, UK; 5 School of Life Sciences, Faculty of Medicine & Health Sciences, The University of Nottingham, Nottingham NG7 2RD, UK; 6 Wolfson Centre for Global Virus Infections, The University of Nottingham, Nottingham NG7 2UH, UK; 7 NIHR Biomedical Research Centre, Nottingham University Hospitals Trust, Nottingham NG7 2UH, UK; 8 Department of Tropical Disease Biology, Liverpool School of Tropical Medicine, Pembroke Place, Liverpool L3 5QA, UK; 9 NIHR Biomedical Research Centre, Oxford University NHS Trusts, Oxford OX3 9DU, UK; 10 Nuffield Department of Medicine, University of Oxford, Oxford OX3 7FZ, UK

## Abstract

Hepatitis C virus (HCV) exhibits significant genetic diversity and is a cause of severe liver complications. The viral envelope glycoproteins E1 and E2, key targets for neutralizing antibodies, are highly variable. To understand how host and viral genetic factors modulate antibody responses, we analyze genetic and antibody binding and neutralization data from 54 patients infected with HCV genotype 3a. We find that host polymorphisms in *IFNL*4 gene (IFNλ4-P70 generating haplotype) are associated with reduced antibody binding. Within the virus, variations at three specific E1/E2 amino acid positions and three N-glycosylation sites (including one site common to both analyses) significantly correlate with antibody binding and/or neutralization sensitivity. Furthermore, greater intra-patient diversity within the E2 hypervariable region 1 is associated with stronger antibody binding. These results identify specific host and viral genetic features that shape humoral immunity against HCV genotype 3a, providing insights crucial for designing broadly effective vaccines.

## Graphical abstract

**Graphical abstract:**
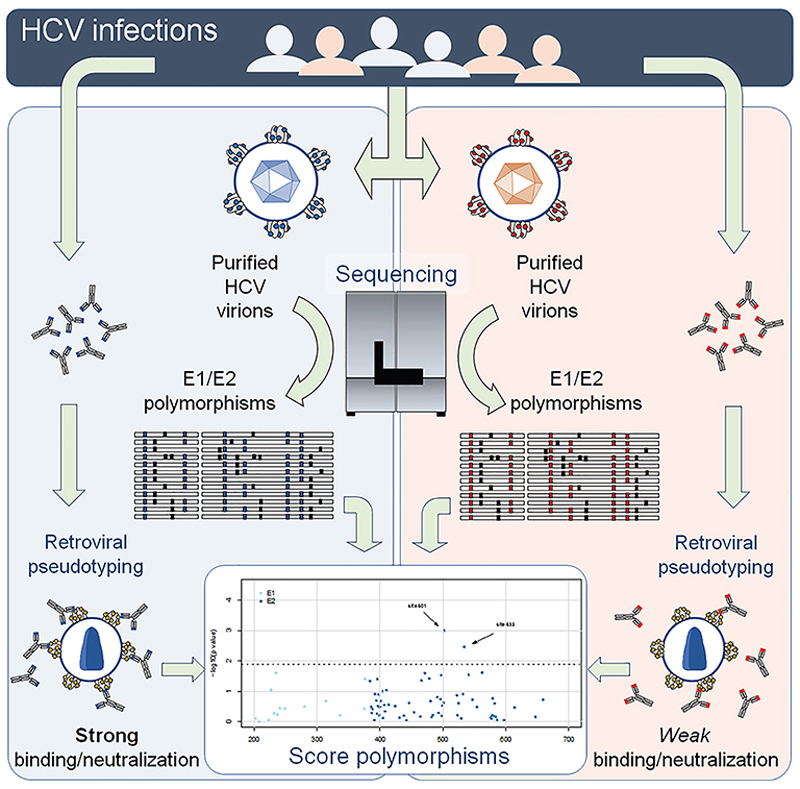

## Introduction

Elimination of the hepatitis C virus (HCV) as a public health concern is a World Health Organization priority to be delivered by 2030. Despite the great success of direct-acting antiviral therapies (DAA),^1^ progress in developing an effective vaccine has been slow.^2^ Virus-neutralizing antibodies target the HCV-encoded E1 and E2 glycoproteins that mediate viral entry.^3,4^ Though the molecular targets of these antibodies are well defined,^2^ the antibody correlates of protection remain unclear. The E2 protein is the major receptor-binding protein that directly engages with the host cell membrane entry factors,^5–9^ while the E1 protein is proposed to stabilize the structure of E2,^10^ and contributes to receptor binding and fusion events that lead to delivery of the virus genome into a cell.^9,11^ The E1/E2 complex possesses a novel structure with E1 performing a “grasping” mechanism to interact with the stem of E2.^10,12^ This structure is consistent with the E2 protein being the receptor binding protein,^13^ which is known to interact with the molecules CD81 and scavenger receptor class B type 1 on the surface of hepatocytes.^6,14^

HCV is highly mutable due to the error-prone nature of genome replication mediated by the NS5B polymerase. The virus displays extensive genome plasticity (reviewed in Echeverria et al.^15^), with individual strains differing by more than 35% of their nucleotide sequence at the extremes of diversity. As a consequence, HCV genome sequences are classified into eight distinct genotypes. Within genotypes, many subtypes have been classified with a maximum of 20%–25% difference between genomes in the same subtype. The most common HCV genotypes causing infection are 1 and 3,^16^ with genotype 1 being highly prevalent in Europe, America, and far eastern countries, and genotype 3 highly prevalent in South Asian countries.^17^ The genotype of the infecting virus influences the specificity of the antibody response to the E1 and E2 glycoproteins,^18^ presenting a challenge for vaccine design.

Understanding the functional consequences of polymorphisms in the viral proteins may provide insights into the development of new antivirals and vaccines. Even minor differences in amino acid sequence can result in considerable functional changes. While the current generation of DAA therapies is effective against all major viral genotypes, some subtypes exhibit inherent drug resistance.^19–22^ HCV genotype 3 presents specific challenges, being associated with faster rates of disease progression compared to other genotypes,^23^ and subtype 3b is inherently more resistant to DAAs.^22^ Genetic variability is not uniform across the virus genome, with extreme diversity exhibited in the E1 and E2 coding genes. This has provided a barrier to the development of a vaccine that can elicit antibodies capable of neutralizing the wide range of *in vivo* HCV isolates.^24–26^ We have previously characterized the antibody neutralization resistance of the major HCV genotypes and subtypes,^18,27,28^ identifying patterns of antibody reactivity associated with specific viral isolates. However, examining the specificity of the antibody response in polyclonal antibody preparations from natural infection is an ongoing challenge.

Glycosylation is a major and crucial co- and post-translational modification of proteins that leads to glycoprotein formation by means of various glycopeptide linkages.^29^ The β-glycosylamine linkage of N-acetylglucosamine to asparagine (N-glycosylation) represents the most widely distributed carbohydrate–peptide bond, and is the most common type of glycosylation, typically occurring at the consensus sequon Asn-X-Ser/Thr (N-X-S/T), where X can be any amino acid except proline. The binding and cleavage of the glycan-amino acid linkage are critical to the folding and biological activity of glycoproteins. Alterations in glycosylation are associated with diverse diseases. The HCV E1 and E2 proteins are extensively glycosylated with N-linked glycans, with up to five sequons on E1 and up to 11 sequons on E2, accounting for approximately one-third of the heterodimer molecular mass.^30^ Despite the high sequence heterogeneity, many of the N-glycosylation sites in the E1 and E2 proteins are conserved among the various HCV genotypes and are important for evasion of antibody binding.^31,32^ However, these carbohydrates provide a target for interactions with the lectins that contribute to the innate immune response.^33,34^

Host genetic variation is one of the factors that drives antibody responses. Genetic variations in the interferon lambda 4 (*IFNL*4) gene are associated with the spontaneous clearance and IFN-based treatment response of HCV infection.^35,36^ Two polymorphisms in the *IFNL4* gene, rs11322783 [ΔG > TT] and rs117648444 [G > A], create four haplotypes,^37^ two that do not produce IFNλ4 protein (TT/G or TT/A: IFNλ4-null) and two that express IFNλ4 protein variants (ΔG/G: IFNλ4-P70 and ΔG/A: IFNλ4-S70). Patients with the IFNλ4-null and IFNλ4-S70 variants exhibit lower hepatic interferon-stimulated gene expression, which is associated with increased viral clearance and response to IFN-based therapy compared to those with the IFNλ4-P70 variant.^38^ Additionally, genetic variation in the HLA region has been reported to be associated with spontaneous clearance of HCV infection.^39^

We employed a genetic screening approach to uncover virus polymorphisms in the HCV E1/E2 coding region linked to anti-E1/E2 antibody responses in patients with HCV genotype 3a infection. Our investigation unveiled three commonly observed amino acid substitutions in the HCV E2 protein that are associated with anti-E1/E2 antibody binding and neutralization sensitivity in an HCV pseudotype-based infection model. Additionally, variants in three N-linked glycosylation sites (one overlapping with an identified amino acid site) were found to be associated with antibody responses. We also investigated the relationship between *IFNL4* gene variants and the antibody response and identified an association between the *IFNL4* haplotype related to production of IFNλ4-P70 and lower levels of antibody response. The identification of polymorphisms and the presence of different N-linked glycosylation motifs in the HCV E1/E2 protein associated with antibody response provide potential targets for vaccine development. In summary, our study suggests that genetic variation in both the virus and the host can impact the potency of the antibody response to HCV infection, and these findings have the potential to inform the development of more effective and personalized HCV vaccines.

## Results

### Cohort description and measurement of HCV-specific antibodies

**Figure 1:**
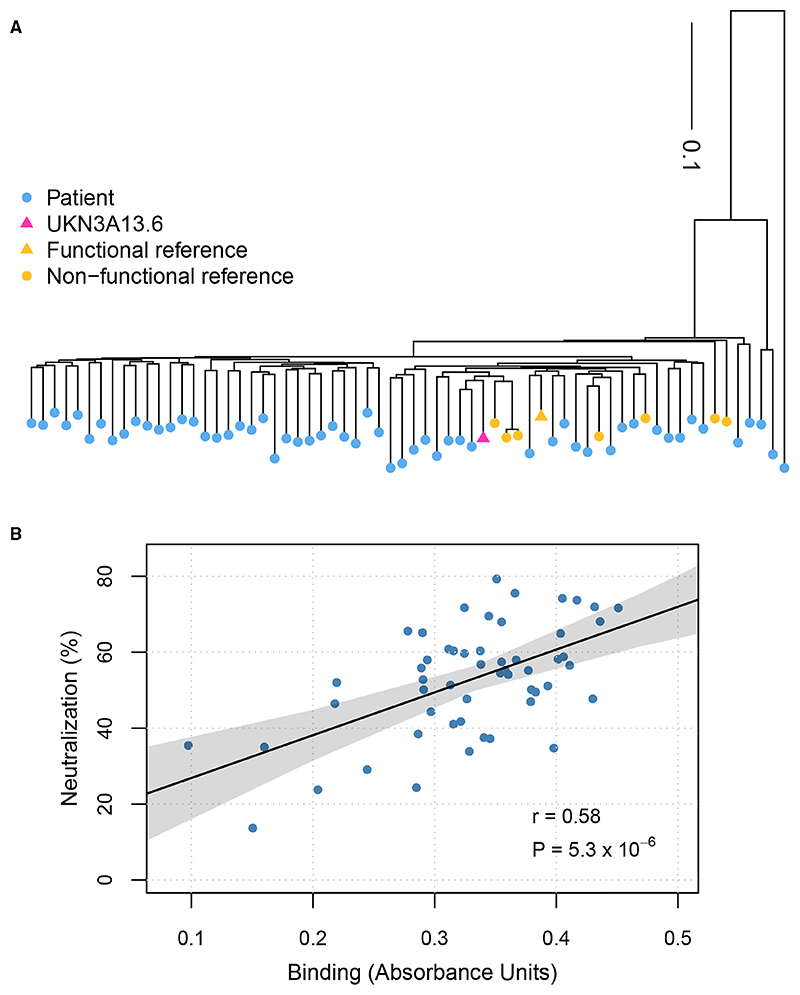
The selection of the reference isolate and the correlation between the binding responses and neutralization sensitivity (A) Maximum likelihood phylogenetic tree showing the relationship between the study isolates (blue) and reference isolates (yellow). Functional and non-functional reference isolates are denoted by triangles and circles, respectively. The chosen reference isolate UKN3A13.6 is highlighted with a red triangle. (B) Correlation between binding and neutralization. The solid black line represents the best-fit linear regression line, where the *p* value for the slope being non-zero is *p =* 5.3 × 10^–6^.

Samples from 60 individuals from the BOSON clinical trial (registration no. NCT01962441) were studied.^40^ Utilizing baseline plasma samples, the polyclonal antibody binding response directed to the full-length E1 and E2 envelope glycoproteins was assessed by ELISA, using an E1/E2 expression construct produced in human HEK293T cells. As a target antigen, a well-characterized clone (UKN3A13.6) was utilized.^41^ This expression clone was previously identified as functional in a retrovirus pseudotype infection assay, confirming the expression of the correctly folded protein able to utilize cellular receptors for specific entry. The sequence of this antigen clustered among our study samples in a maximum likelihood phylogenetic tree (Figure 1A) based on E1 and E2-encoding nucleotide sequences. Initial experiments titrated all sera against the reference UKN3A13.6 antigen (Figure S1). Additionally, we used the baseline plasma samples to evaluate the neutralization sensitivity of the antibody responses in each individual using retroviruses pseudotyped with an identical full-length E1/E2 construct representing UKN3A13.6. The ELISA and neutralization assays were reproducible with mean coefficient of variations of 8.5% and 14.4%, respectively. Entire consensus genome sequences for HCV were generated for 57 of the infections. Of these, 54 individuals were infected with HCV genotype 3a (gt3a), and of the remaining three, two were infected with gt3b and one with gt2b. To minimize the impact of virus genetic heterogeneity, we focused on samples from 54 patients infected with gt3a viruses. We also examined the relationship between host *IFNL4* gene haplotypes and HLA alleles with antibody responses in the context of chronic HCV infection.

After performing antibody binding and neutralization assays, we used a linear regression analysis to determine whether binding and neutralization were associated with the number of amino acid differences between the reference UKN3A13.6 antigen and the HCV consensus sequences of the study samples. No significant associations between binding and the number of amino acid differences (*p* = 0.36, Figure S2A) or neutralization and the number of amino acid differences (*p* = 0.4, Figure S2B) were found. Moreover, we observed a significant positive correlation between binding and neutralization (r = 0.58, *p* = 5.3 × 10^–6^, Figure 1B).

### Non-genetic factors associated with antibody responses

**Figure 2:**
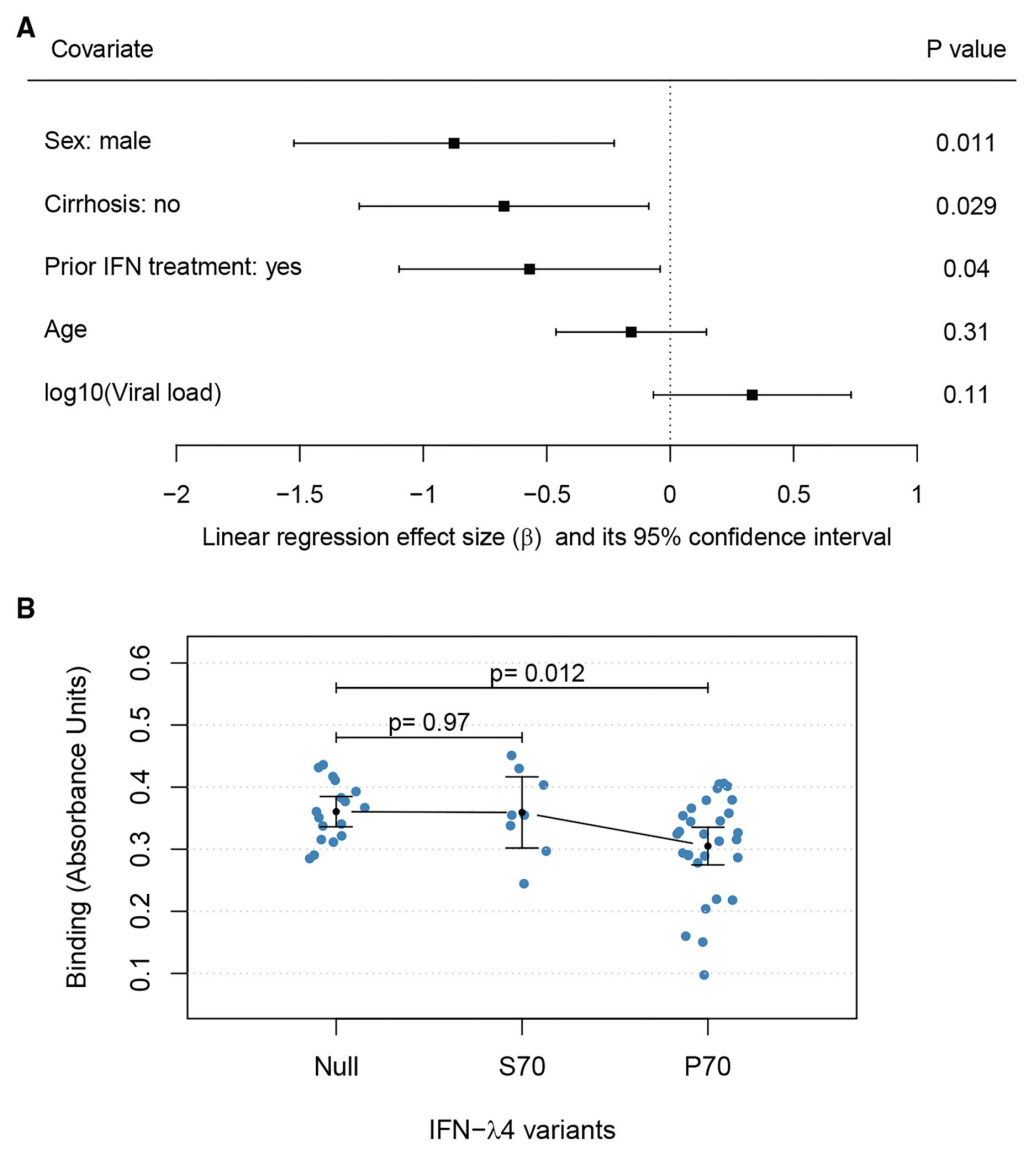
The impact of host genetic and non-genetic factors on antibody binding response in HCV infection (A) Forest plot of the effect sizes and their confidence intervals (CIs) for non-genetic factors tested against binding. The squares show the linear regression estimated effect sizes for each covariate, and the lines show their 95% CI. The *p* values for each covariate are shown on the right (*n =* 54). (B) Binding stratified by the host *IFNL4* gene haplotypes linked to the three protein variants (IFNλ4-null, IFNλ4-S70, and IFNλ4-P70). We employed a dominant genetic model, where individuals with one or two copies of IFNλ4-P70 were classified as IFNλ4-P70, while individuals with two copies of IFNλ4-S70 or one copy of IFNλ4-S70 and one copy of IFNλ4-null were classified as IFNλ4-S70. The black dots and lines indicate the mean and its 95% CI for each group. The *p* values for the difference in mean relative to the IFNλ4-null were calculated using linear regression (*p*_S70_ = 0.97 and *p*_P70_ = 0.012).

In a multivariable linear regression analysis, we investigated the association between antibody response and several factors, including sex, cirrhosis status, prior interferon treatment, age, and log_10_ viral load (Figure 2A). Patient’s sex had the largest impact on the antibody binding response, where males had lower anti-E1/E2 binding levels than females (*p* = 0.011). Cirrhosis and previous interferon treatment status were also associated with binding, with patients without cirrhosis or with previous interferon treatment displaying lower antibody binding responses (*p*_cirrhosis_ = 0.029 and *p*_prior-treatment_ = 0.04). Higher viral load was associated with higher binding, but this effect was not statistically significant (*p*_viral-load_ = 0.11). We also tested the association between neutralization and the same factors using linear regression (Figure S3A). Prior interferon treatment was marginally associated with lower levels of neutralization sensitivity (*p* = 0.044). Male sex and absence of cirrhosis were associated with lower neutralization sensitivity, but the effects were not statistically significant (*p*_male_ = 0.089 and *p*_prior-reatment_ = 0.1, Figure S3A).

### Host genetic factors associated with antibody responses

Since *IFNL4* gene haplotypes are highly associated with HCV spontaneous clearance and IFN-based treatment response, we hypothesized they may also be associated with antibody responses. We examined the association between the three haplotypes linked to the three protein variants (IFNλ4-null, IFNλ4-S70, and IFNλ4-P70) and antibody responses (Figures 2B; S3B). We employed a dominant genetic model, where individuals with one or two copies of IFNλ4-P70 were classified as IFNλ4-P70, while individuals with two copies of IFNλ4-S70 or one copy of IFNλ4-S70 and one copy of IFNλ4-null were classified as IFNλ4-S70. Patients classified as IFNλ4-P70 were observed to have significantly lower antibody binding than patients with IFNλ4-null (*p* = 0.012) in regression analysis. However, antibody binding levels were similar in patients with IFNλ4-S70 and IFNλ4-Null variants (*p* = 0.97). We observed a modest reduction in antibody neutralization sensitivity in IFNλ4-P70 individuals compared to the other two groups, although the result was not statistically significant (Figure S3B). We also investigated the association between HLA alleles and antibody response in HCV infection (Tables S1 and S2). HLA-A*03:01 allele was nominally associated with a reduction in antibody neutralization sensitivity (*p* = 0.023), but after multiple testing correction, the effect was not statistically significant.

### Virus genetic factors associated with antibody responses

**Figure 3:**
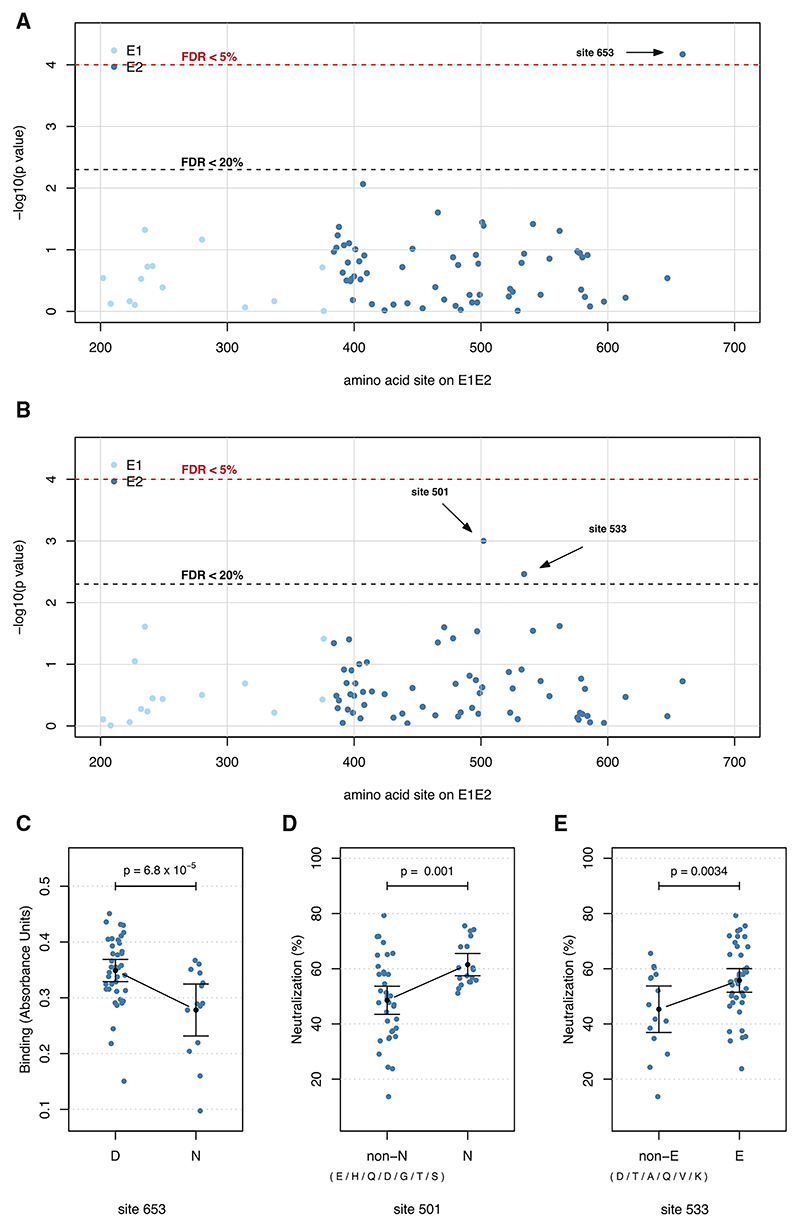
Association between HCV amino acid polymorphisms in E1 and E2 proteins and antibody response (A) Association with binding response. Site 653 (relative to the H77 polyprotein) is significantly associated with antibody binding response at 5% false discovery rate (FDR). (B) Association with antibody neutralization sensitivity. Sites 501 and 533 are significantly associated with neutralization sensitivity at 20% FDR. (A and B) The red and black dashed lines represent FDR thresholds of 5% and 20%, respectively. (C) Site 653 is associated with the binding response. In patients whose virus carried aspartic acid (D) at this site, the antibody binding response was higher than in patients whose virus carried asparagine (N) at this site. *p =* 6.8 × 10^–5^, calculated using linear regression. (D) Site 501 is associated with neutralization sensitivity. Asparagine (N) at site 501 was the most associated residue. In patients whose virus carried asparagine (N) at this site, the antibody neutralization sensitivity was higher than in patients whose virus carried other amino acids at this site. *p =* 0.001, calculated using linear regression. The amino acids listed in the non-N group are ordered in increasing frequency. (E) Site 533 is associated with neutralization sensitivity. Glutamic acid (E) at site 533 was the most associated residue. In patients whose virus carried glutamic acid (E) at this site, the antibody neutralization sensitivity was higher than in patients whose virus carried other amino acids at this site. *p =* 0.0034, calculated using linear regression. The amino acids listed in the non-E group are ordered in increasing frequency. In (C)– (E), the black dots and lines indicate the mean and 95% CI for each group.

We tested for associations between consensus amino acid variants in E1 and E2 proteins and antibody response using linear regression analysis. Sex, cirrhosis status, prior IFN-based treatment, log10 of baseline viral load, and *IFNL4* haplotypes were included as covariates to minimize the possible confounding effects. In our analysis, we used binding and neutralization data as response variables and the presence or absence of amino acid residues in polymorphic sites of E1 and E2 glycoproteins as explanatory variables. We only tested residues that were present in at least ten isolates, which resulted in testing 123 residues (at 77 sites) in each of the assays (Tables S3 and S4). At a false-discovery rate (FDR) of 5%, we identified one viral polymorphism—site 653 in E2 (numbering relative to H77 polyprotein)—that was significantly associated with antibody binding (Figure 3A). Increasing the FDR to 20% did not lead to any new findings. For neutralization sensitivity, no sites reached significance at 5% FDR; however, at 20% FDR, we observed two sites (sites 501 and 533 in E2) associated with neutralization sensitivity (Figure 3B). At site 653 (associated with 5% FDR, the most common sequence surrounding the site 653 RGERC**D**IEDRD), asparagine (N) was associated with reduced binding relative to aspartic acid (D) (Figure 3C, *p* = 6.8 × 10^–5^). However, this site was not associated with antibody neutralization (*p* = 0.19, Figure S4A). Amino acid variations at sites 501 and 533 in the E2 protein (detected at 20% FDR) were associated with antibody neutralization sensitivity, where asparagine (N) at site 501 was associated with higher levels of neutralization sensitivity relative to other residues (*p* = 0.001, Figure 3D, the most common sequence surrounding the site 501 IVPAL**N**VCGPV). At site 533, possessing a glutamic acid (E) residue was associated with higher neutralization sensitivity (*p* = 0.0034) compared to other residues at this site (Figure 3E, the most common sequence surrounding site 533 TWGEN**E**TDVFL). The impact of these residues on binding was consistent with their impact on neutralization sensitivity; however, the effects were non-significant (*p*_501_ = 0.067 and *p*_533_ = 0.053, Figures S4B and S4C). These three sites are highly variable in wild-type gt3a isolates. To see the frequency of the amino acids at these three associated sites in the full BOSON dataset (*n* = 507), we calculated the frequency of all the observed amino acids as shown in Table S5.

To experimentally assess the phenotypic impact of the polymorphisms associated with neutralization, we evaluated the impact of the relevant polymorphisms (T501N and E533K) on viral fitness in our HCV pseudotype model. However, both polymorphisms were associated with extremely low levels of infectivity, and as such it was not possible to assess their relevance to antibody-mediated neutralization. This highlights the importance of these sites in virus for cell entry (Figure S5).

### Virus glycosylation is associated with antibody responses

**Figure 4:**
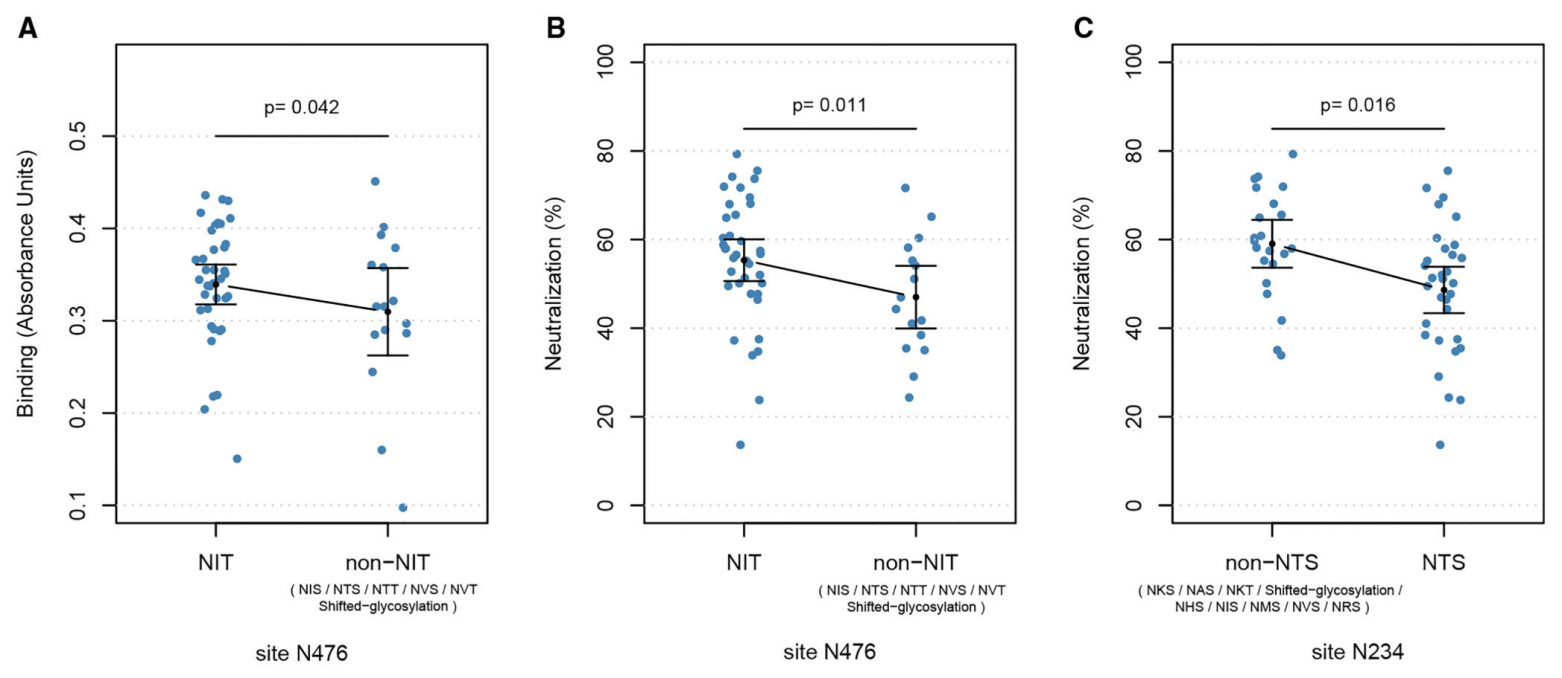
Association between variants in HCV glycosylation sites in E1 and E2 proteins and antibody response (A) Site N476 is significantly associated with antibody binding response at 20% FDR. In patients whose virus carried NIT motif at this location, the antibody binding response was higher than in patients whose virus carried non-NIT motifs (*p =* 0.042 from linear regression). The motifs listed in the non-NIT group are ordered in decreasing frequency. (B) Site N476 is significantly associated with neutralization sensitivity at 20% FDR. In patients whose virus carried NIT motif at this location, the antibody neutralization sensitivity was higher than in patients whose virus carried non-NIT motifs (*p =* 0.011 from linear regression). The motifs listed in the non-NIT group are ordered in decreasing frequency. (C) Site N234 is significantly associated with neutralization sensitivity at 20% FDR. In patients whose virus carried NTS motif at this location, the antibody neutralization sensitivity was lower than in patients whose virus carried non-NTS motifs (*p =* 0.016 from linear regression). The motifs listed in the non-NTS group are ordered in decreasing frequency. The black dots and lines indicate the mean and 95% CI for each group.

We conducted a linear regression analysis to test for the association between the changes in glycosylation motifs in E1 and E2 and antibody responses. Antibody neutralization and binding were used as the outcome variables, while the presence or absence of different glycosylation sequon motifs at each glycosylation site was used as the exposure variable. To control the possible confounding factors, sex, cirrhosis status, prior IFN-based treatment, *IFNL4* haplotypes, and log10 of baseline viral load were included as covariates in the model. Most of the sequences (48/54) carried 15 glycosylated sites in their E1/E2 proteins (Table S6), which are characteristic of genotype 3 viruses. We observed that at some of the sites, the glycosylation motifs occasionally shifted by one, two, or three positions (Figure S6). Of the 15 conserved glycosylated sites, six sites carried at least two different motifs with frequencies greater than 10. We only performed the linear regression at these six sites. As the number of tests was limited, we combined the *p* values from the binding and neutralization tests to calculate the FDR. We observed no significant associations at a 5% FDR. After increasing the threshold to 20%, we found that motifs at three glycosylation sites were associated with binding and/or neutralization (Table S7). Respectively, motif NIT at site N476 (in E2 protein, numbering relative to H77 polyprotein) was significantly associated with higher levels of binding and neutralization response compared to non-NIT motifs (Figure 4A *p*_*binding*_ = 0.042 and 4B *p*_*neutralization* *=*_ 0.011). Additionally, the NTS motif at site N234 (in E1 protein, numbering relative to H77 polyprotein) was found to be associated with reduced neutralization sensitivity, relative to non-NTS motifs (Figure 4C, *p* = 0.016). The NTS motif at this site was associated with lower binding response; however, the effect was not statistically significant (Figure S7, *p* = 0.095). Lastly, the NET motif at N532 (in E2 protein) was associated with increased neutralization sensitivity versus non-NET motifs. This N532 sequon notably includes amino acid position 533, where glutamic acid (E), part of the NET motif, independently associated with higher neutralization sensitivity in our separate amino acid analysis (Figures 3B and 3E).

### The association of intra-patient viral nucleotide diversity and antibody responses

**Figure 5:**
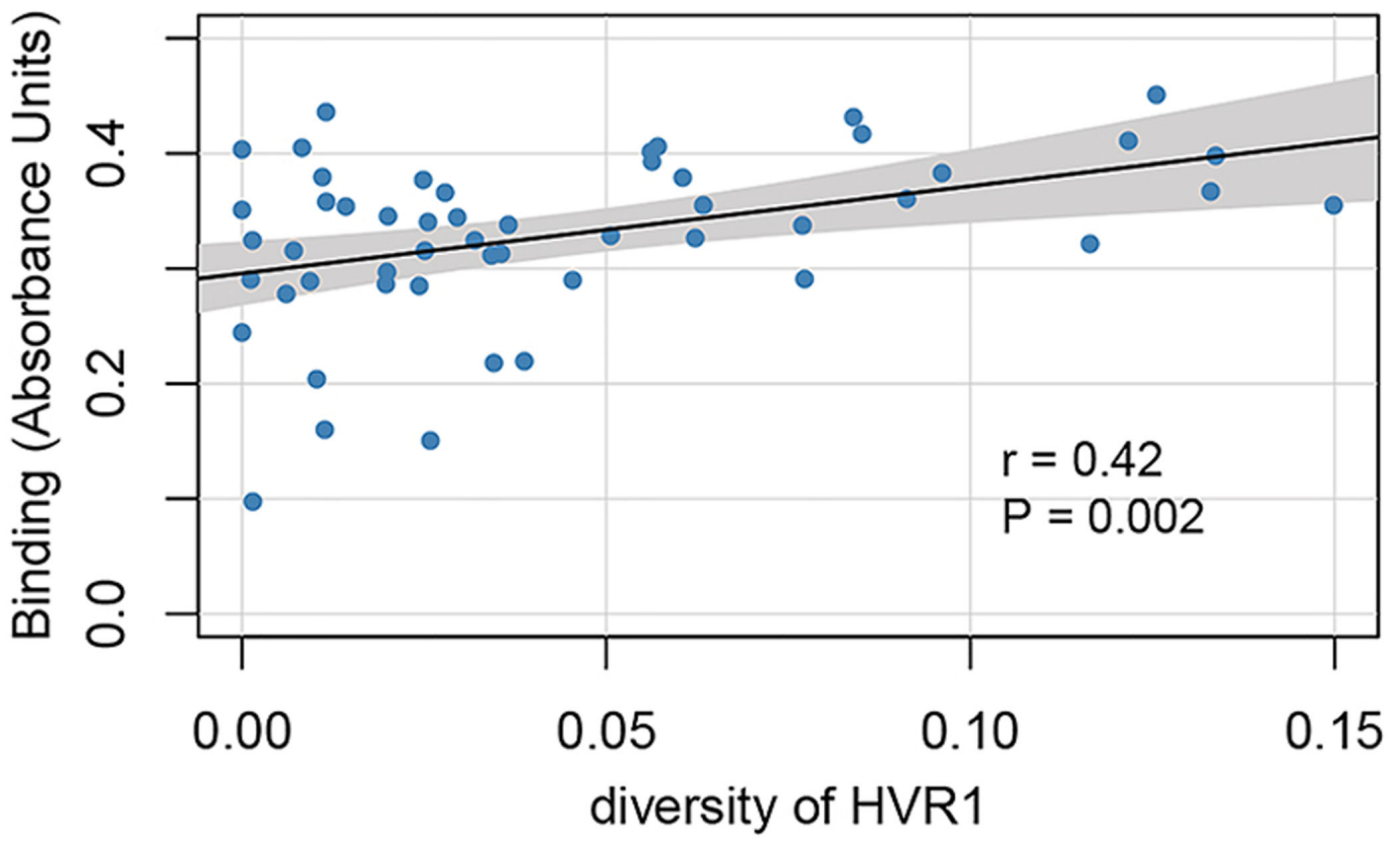
Correlation between intra-patient viral nucleotide diversity in HVR1 and antibody binding *x* axis indicates the mean nucleotide diversity in HVR 1, and the *y* axis is the antibody binding response. The black line shows the best-fit linear regression line, and gray area indicates its 95% CI. *p* = 0.0016 calculated from linear regression.

We investigated the relationship between intra-patient viral diversity at the nucleotide level and antibody responses. Nucleotide diversity, calculated from next-generation sequencing reads, was used to measure intra-patient viral diversity. We used linear regression with binding as the outcome variable and intra-patient diversity as the exposure variable to test for associations between antibody responses and the mean nucleotide diversity across the entire genome, in E1 and E2 genes (Figure S8A), and the hypervariable region 1 (HVR1). We found an association between intra-patient viral diversity in HVR1 and binding response (Figure 5, *p* = 0.0016, r = 0.42), where an increase in intra-patient HVR1 diversity was associated with greater binding. However, there was no significant association between intra-patient viral diversity and neutralization sensitivity (Figure S8B).

### Mapping antibody-associated polymorphisms to surface-exposed E1/E2 regions

To evaluate whether polymorphisms associated with antibody responses localize to structurally accessible and immunologically relevant regions of the HCV E1/E2 glycoproteins, we analyzed both sequence variability and protein structure using genotype 3a sequences from our cohort. A multiple sequence alignment of E1/E2 sequences was generated and analyzed using ConSurf^42^ to compute residue-specific evolutionary conservation scores. These scores were then mapped onto the surface of the recently resolved genotype 3a E1/E2 heterodimer structure (PDB ID: 8RJJ),^12^ allowing visualization of variability across the three-dimensional conformation of the glycoprotein.

This analysis revealed that the polymorphism at residue 653—associated with differential antibody binding—resides on a highly conserved region of the E2 surface (Figure S10A). In contrast, glycosylation sites at positions 234 (in E1), 476, and 533 (in E2), along with amino acid residue 501 (in E2), all of which were associated with antibody neutralization sensitivity, mapped to regions exhibiting elevated sequence variability (Figure S10A). These findings suggest that antibody-binding epitopes under limited immune pressure (e.g., residue 653) tend to be structurally conserved, whereas sites involved in neutralization are more variable and likely subject to immune-driven selection. To complement these structural insights, we visualized sequence variation across the linear E1/E2 protein, highlighting major peaks in amino acid diversity that correspond to known immunogenic domains, including HVR1, HVR2, and the inter-genotypic variable region (Figure S10B).

**Figure 6:**
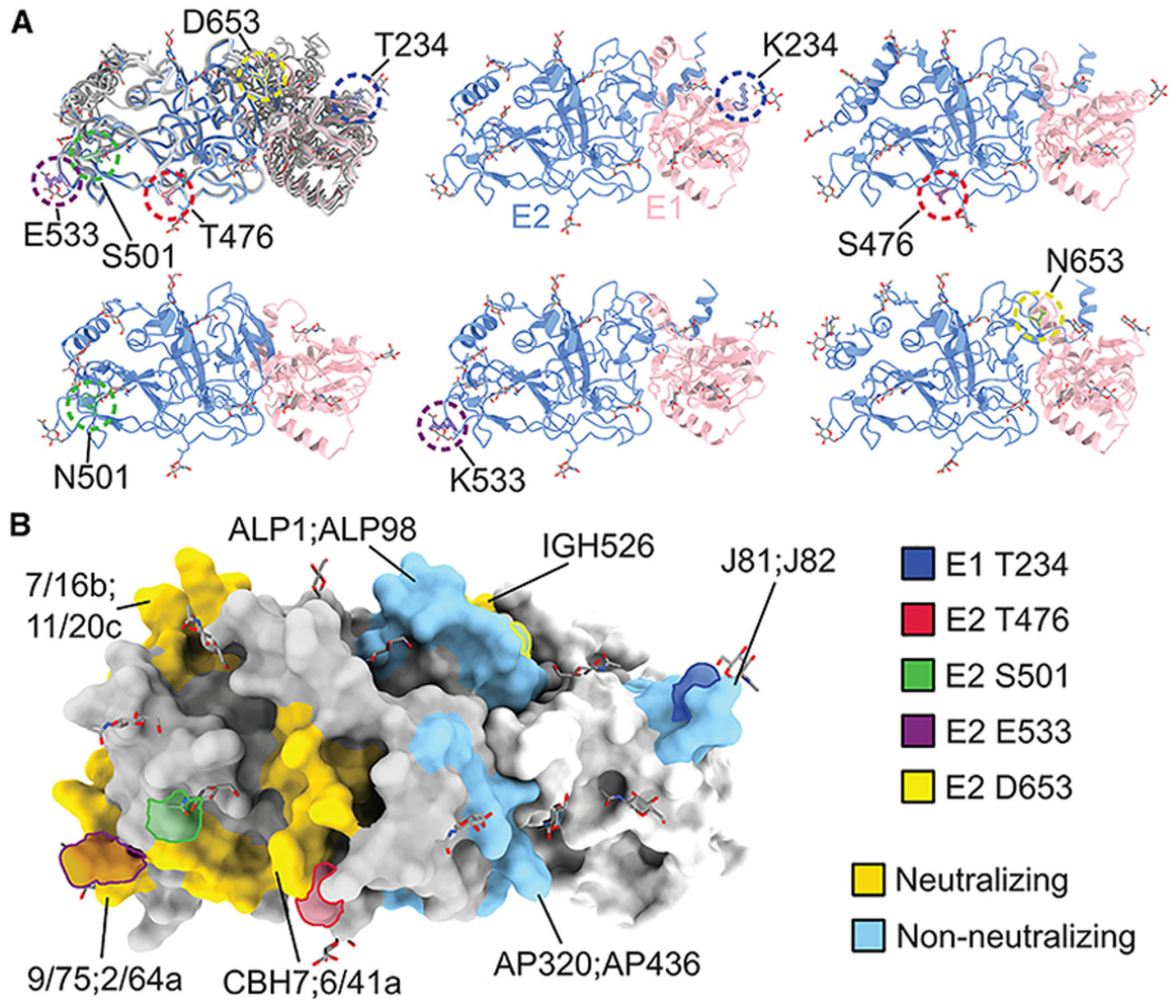
Structural localization and epitope context of antibody-associated polymorphisms in HCV E1/E2 (A) Structural models of the HCV E1/E2 ectodomain were generated using AlphaFold3 based on consensus genotype 3a sequences. Models were created for the consensus sequence (E1 in pink and E2 in blue) as well as for five individual variants identified in this study: T234K (E1), T476S, S501N, E533K, and D653N (all in E2). Overlaying each mutated structure (shown in gray) onto the consensus model revealed no major conformational changes, suggesting that these polymorphisms are unlikely to induce large-scale structural rearrangements. However, given that Alphafold3 is unlikely to be sensitive to localized structural differences, we cannot preclude the possibility that the observed polymorphisms introduce subtle structural effects. (B) Surface rendering of the HCV E1/E2 heterodimer in the same orientation as in (A), with E1 shown in white and E2 in gray. Polymorphic sites are indicated as colored outlines corresponding to the residue-specific key. Known neutralizing antibody epitopes (yellow) and non-neutralizing epitopes (blue) are mapped onto the surface. Polymorphisms at sites 501, E533, and 476 (E2) overlap with the footprints of neutralizing monoclonal antibodies (e.g., 9/75, 2/64a, CBH7, 6/41a, 7/16b, and 11/20c), while polymorphisms at 234 (E1) and 653 (E2) lie within the regions targeted by non-neutralizing antibodies (e.g., ALP1, ALP98, J81, and J82). These spatial overlaps support the immunological relevance of the identified sites. The color key for polymorphic sites is as follows: blue outline: E1 234 (neutralization-associated); red outline: E2 476; green outline: E2 501; purple outline: E2 533; yellow outline: E2 653 (binding-associated); gold shading: neutralizing antibody epitopes; and blue shading: non-neutralizing antibody epitopes.

To explore the potential structural impact of the identified polymorphisms, we generated AlphaFold3-based structural models of E1/E2 incorporating the non-reference residues for each of the five key polymorphic sites (Figure 6A). While subtle local rearrangements cannot be ruled out, these variants were not predicted to induce major conformational changes relative to the reference structure. This supports the interpretation that the observed differences in antibody binding and neutralization are more likely driven by epitope-specific surface alterations or glycan structure or accessibility rather than gross structural remodeling. Finally, we examined the spatial overlap between the identified polymorphisms and known monoclonal antibody (mAb) epitopes (Figure 6B). Mapping of epitope footprints revealed that three of the four polymorphic sites associated with neutralization (501, 533, and 476 in E2) lie within or are proximal to the binding sites of well-characterized neutralizing mAbs, including 9/75, 2/64a, CBH7, and 6/41a. E1 site 234, also associated with neutralization, overlaps epitopes recognized by mAbs J81 and J82. The only polymorphism associated with differential antibody binding, 653 (in E2), overlaps with the non-neutralizing antibody ALP1 and is adjacent to others such as the non-neutralizing antibody ALP98 and the neutralizing antibody IGH526.^43^ These spatial overlaps support the immunological relevance of the identified sites and suggest that natural polymorphisms at these positions may alter antigenicity through disruption or masking of key antibody contact points and support their potential relevance as targets in vaccine design.

## Discussion

We report the analysis of associations between amino acid variation in HCV E1 and E2 protein sequences and host antibody responses in a well-characterized patient cohort. We also examined the impact of host *IFNL4* genetic variation, HLA alleles, and clinical phenotypes on anti-E1/E2 responses. Our analysis distinguished between antibodies that bind E1/E2 glycoproteins and those possessing neutralizing activity capable of limiting HCV pseudoparticle (HCVpp) infection. The non-neutralizing antibodies may have other antiviral functions *in vivo*,^44^ such as antibody-dependent cellular cytotoxicity (ADCC), which is not measured in our neutralization assay. We used linear regression to test for association between virus amino acid polymorphisms in E1 and E2 and antibody responses, identifying three amino acid sites in the gt3a E2 protein that impact the antibody responses. Additionally, distinct *N*-glycan motifs present at three specific *N*-glycosylation sites (one overlapping with an identified amino acid site) in E1/E2 were associated with antibody binding and/or neutralization. Furthermore, we found that intra-patient virus nucleotide diversity in the HVR1 region was associated with host antibody binding against E1/E2. We also observed that host *IFNL4* gene polymorphisms were associated with antibody responses as well as other factors such as sex and cirrhosis status of the patient.

Three sites in the E2 protein possessed amino acid polymorphisms associated with differential anti-E1/E2 antibody responses in gt3a virus-infected patients. Among these sites, the strongest association was at site 653, which was significant at 5% FDR. The asparagine (N) and aspartic acid (D) residues at position 653 were found to be associated with antibody binding, which is part of a region previously reported to be targeted by non-neutralizing antibodies.^45^ This polymorphism is located in a region of the E2 stem (Figure S9) known to be immunogenic in experimental immunizations, with murine mAbs ALP11, AP266, ALP1, ALP98, and H52 specifically targeting this region.^46^ Our reference E1/E2 antigen utilized in the assays possessed an aspartic acid residue at this site, and we observed higher binding in patients whose infecting virus carried aspartic acid compared to those with asparagine at this site. Structural modeling using AlphaFold3 suggested that the D653N substitution does not induce large-scale conformational changes in the E1/E2 heterodimer. However, given its surface accessibility and overlap with non-neutralizing epitope footprints such as ALP1 and ALP98, it is plausible that local side-chain variation or residue-specific recognition underlies the observed association, consistent with a model of variant-specific antibody binding shaped by minor surface alterations rather than global conformational shifts.

The amino acid polymorphisms observed to influence the neutralizing antibody response (at 20% FDR) located at positions 501 and 533 are presented at the neutralizing face of the E2 ectodomain^47^ overlapping with the CD81 binding site. Site 533 is located in a relatively conserved region (533–535) critical for CD81 receptor interaction.^48^ The majority of viruses in our study possessed a glutamic acid (E) at this site, although we observed several other residues at this site, including K, V, Q, A, T, and D (Table S5). The reference antigen encoded a glutamic acid residue at site 533, and we observed higher levels of neutralization sensitivity in plasma from patients whose virus possessed a glutamic acid at this site relative to other residues. Structural mapping further revealed that site 533 overlaps with the epitope footprints of several broadly neutralizing mAbs, including 9/75 aa and 6/44a (Figure 6), supporting its immunological relevance. Site 533 is also part of the N-linked glycosylation motif starting at N532; concordantly, our glycosylation analysis showed that the NET motif associated with increased neutralization sensitivity compared to non-NET motifs, potentially due to distinct glycosylation efficiencies or glycan structures impacting antibody recognition.

Regarding site 501, its proximity to the CD81 binding site in the tertiary structure of E2 (Figure S9) suggests that substitutions (e. g., N to other residues) might alter the presentation of conformational neutralization epitopes without disrupting receptor binding. Indeed, mutants with the Q501A or S501A substitutions at this site have been implicated to influence monoclonal antibody (mAb) binding,^49,50^ and the N501S mutation altered mAb binding despite having little effect on virus entry.^51^ Studies utilizing antibody production from human antibody libraries^52–54^ and individual human B cells^55,56^ have demonstrated that, unlike experimental immunization, natural infection favors the production of conformation-dependent anti-E2 responses. It is likely that our assays, which measure binding of both conformation-dependent and -independent antibodies, may be influenced by individual or combinations of key polymorphic sites. Structural predictions using AlphaFold3 indicated that the N501S substitution does not introduce large-scale conformational shifts, but given the residue’s surface accessibility and epitope overlap, even minor side-chain alterations could affect antibody recognition.

When individual glycosylation sequons were analyzed in the same manner as individual amino acids, it was revealed that three sites (N234, N476, and N532 at 20% FDR) were associated with the antibody response in this patient cohort. Beyond the NET sequon at site N532, which was associated with higher levels of antibody neutralization sensitivity, sequon variations at N234 and N476 were also associated with antibody response. N234 and N476 were previously implicated in resistance to neutralization, where the removal of these N-linked glycans in HCV gt1a mutants individually reduced sensitivity to polyclonal serum antibodies.^57^ It is plausible that the alternative sequons identified here modify the efficiency of glycosylation or the resulting glycan structure at these sites, impacting the sensitivity to neutralization. Different NX(S/T) sequons are known to be glycosylated with varying efficiencies,^58^ a phenomenon demonstrated for other viral glycoproteins,^59^ and, as such, it is plausible that selection of different sequons during the evolution of different virus strains will be associated with different carbohydrate modifications. Further investigations are required to determine if differences in the patterns of glycosylation at sites 234, 476, and 532 influence the efficiency or structure of carbohydrate modifications, as predicted for other virus glycoproteins.^60^

The observation that residues and glycosylation motifs associated with antibody neutralization sensitivity (234, 476, 501, and 533) map to regions of high sequence variability in our cohort, as visualized on the genotype 3a E1/E2 heterodimer structure (Figure S10), supports the notion that these sites are subject to immune selection pressure. This aligns with previous findings showing that viral escape from neutralizing antibodies during acute HCV infection is often mediated by amino acid substitutions at exposed, immunogenic sites.^61^ Our findings extend this concept to chronic infection, suggesting that sustained antibody pressure may continue to shape viral sequence diversity at these neutralization-sensitive loci. In contrast, the polymorphism at residue 653 (associated with binding but not neutralization) resides in a more conserved region, implying it may not be under the same selective pressure. This divergence highlights that potentially neutralizing, but not necessarily binding, antibody responses are key drivers of antigenic variation in E1/E2 during natural infection.

Our analyses revealed instances, particularly concerning variations in amino acids and glycosylation motifs, where patient sera exhibited stronger binding or neutralization against reference antigens that shared the same variant found in the patient’s infecting virus. While potentially viewed as an experimental confounder, we interpret this concordance as strong evidence supporting a key conclusion: specific variations in E1/E2 elicit variant-specific antibody responses during natural infection, characterized by preferential recognition of homologous epitopes. We propose that this reflects the host developing tailored humoral immunity specific to the infecting viral variant, underscoring the functional impact of viral diversity on antibody recognition and neutralization. This finding has direct implications for HCV vaccine development. It highlights the challenge posed by viral diversity and demonstrates that antibodies elicited by one viral variant may be less effective against others even within the same virus subtype. Therefore, designing a broadly effective HCV vaccine likely requires strategies that can induce immune responses capable of overcoming this variant-specific recognition, targeting conserved epitopes or utilizing multi-valent formulations to cover key antigenic variations observed in circulating strains.

We also investigated intra-patient viral diversity and observed a significant association between HVR1 nucleotide diversity and antibody binding response. The causal direction is difficult to determine, as a higher antibody response could result in more selective pressure on the virus, encouraging it to develop escape mutations. These mutations, in turn, could lead to greater viral genetic diversity. Alternatively, higher viral sequence diversity could lead to a larger antibody response targeting multiple viruses.

In addition to the virus genetic variations, we showed that host *IFNL4* activity may shape the antibody response. Previous studies have shown that IFNλ4 -P70 and IFNλ4 -S70 variants of IFNλ4 protein have distinct phenotypes both *in vivo* and *in vitro*.^37,62^ Using the genome-wide genotyping data, we inferred haplotypes consisting of rs11322783 [ΔG>TT] and rs117648444 [G>A] variants of *IFNL4* SNPs in our cohort. Assuming a dominant effect (IFNλ4-P70 > IFNλ4-S70 > IFNλ4-null), we observed that individuals with IFNλ4-P70 variant had significantly lower levels of antibody binding response relative to individuals with IFNλ4-S70 and IFNλ4-null variants. We observed the same trend for neutralization sensitivity, but the effects were not statistically significant. The IFNλ4-null variant (CC genotype at SNP rs12979860) was previously reported to associate with higher viral loads, increased spontaneous clearance, improved response to interferon treatment, and lower hepatic interferon-stimulated gene (ISG) expression. Our observation of higher anti-E1/E2 antibody levels in individuals with the IFNλ4 -null variant suggests a potential mechanism, while individuals with the IFNλ4-P70 variant may experience lower viral loads due to higher hepatic ISG expression (which exert antiviral activity); IFNλ4 -P70 itself may dampen the adaptive humoral immune response, potentially contributing to lower rates of spontaneous clearance and treatment response. A potential mechanism for the observed link between *IFNL4* polymorphisms and antibody response may involve ER stress-mediated impairment of major histocompatibility complex class II (MHC class II) presentation, affecting T helper cell function and therefore altering HCV-specific antibody responses.^63^ These findings suggest a role for host *IFNL4* genetic variation in modulating the antibody response to HCV infection.

In conclusion, we provide a detailed investigation of the impact of genetic variation in HCV and in the host *IFNL4* gene on humoral immune responses. We used a hypothesis-free approach and investigated all amino acid polymorphisms in HCV E1 and E2 proteins and discovered three sites associated with antibody responses. We also observed that changes in glycosylation motifs in three sites (including one site common to both analyses) were associated with antibody responses. We also discovered that individuals with IFNλ4-P70 variants have a lower level of antibody response. These observations suggest that both the host genetic background and the virus strain could drive the humoral immune response and together determine the outcome of infection and its control over time. Given that antibody responses are a critical component of most vaccine-induced immunity, our results suggest that individuals with the IFNλ4-P70 variant may exhibit lower responses to an HCV vaccine, underscoring the need for tailored vaccine strategies. Moreover, our discovery that naturally occurring amino acid polymorphisms within a single HCV subtype (gt3a) impact neutralizing antibody responses emphasizes the importance of considering such variations when designing a broadly protective, pan-genotypic HCV vaccine. Such vaccines must address naturally occurring polymorphisms to enhance cross-reactivity against diverse variants and elicit robust humoral immune responses. Finally, the methodology employed in this study is broadly applicable to other viral infections, offering a framework to design vaccines that more effectively elicit immune responses by accounting for both host and viral genetic variability.

### Limitations of the study

While our study provides valuable insights into the interplay between host/viral genetics and antibody responses in HCV genotype 3a infection, several limitations should be acknowledged. First, our analyses relied on specific *in vitro* assays to assess antibody function. Although HCVpp is widely used and provides reproducible neutralization data in our hands,^3,18,51^ it represents a surrogate system. Furthermore, neutralization was assessed exclusively against the UKN3A13.6 patient-derived E1/E2 sequence.^41^ This clone was selected for its central location among our study sequence and its known robust infectivity in HCVpp assays,^27,28,41,64,65^ a trait uncommon among genotype 3a isolates, only about 5% of which yield functional HCVpp in our investigations.^66^ Consequently, neutralization potency against this single strain may not fully reflect the breadth of activity against diverse circulating genotype 3a variants. Similarly, our binding assays used intracellularly expressed E1/E2 captured via Galanthus nivalis agglutinin (GNA)-ELISA. While this method enriches for correctly folded heterodimers with high-mannose glycans resembling those on authentic virions,^67^ potential variations in glycoforms or conformations might still exist. Additionally, our assays do not capture other potentially relevant antibody functions like ADCC. Furthermore, the study design has inherent limitations. With a cohort size of 54 patients, statistical power may be limited for detecting associations with rarer genetic variants or for subgroup analyses. The cross-sectional nature of the analysis captures associations at a specific time point but cannot resolve the temporal dynamics of viral evolution and antibody responses within individuals. While we controlled for several key covariates, the possibility of unmeasured confounding factors (host or viral) influencing the observed associations remains. Finally, this study employed an exploratory statistical approach using a 20% FDR threshold for identifying associations between genetic variants and antibody responses. This threshold prioritizes sensitivity for hypothesis generation but inherently increases the risk of false positive findings. Therefore, while highlighting promising candidates, these findings require independent validation in larger cohorts before definitive biological conclusions can be drawn. Statistical significance alone does not guarantee biological effect, emphasizing the need for replication and further functional studies.

## Resource Availability

### Lead contact

Requests for further information, resources, and reagents should be directed to and will be fulfilled by the lead contact, M. Azim Ansari (azim.ansari@ndm.ox.ac.uk).

### Materials availability

Plasmids encoding the genotype 3 E1/E2 expression constructs, including single-point mutants generated during this project, are freely available by request from corresponding author Alexander W. Tarr (alex.tarr@nottingham.ac.uk).

## Star★Methods

Detailed methods are provided in the online version of this paper and include the following:

- KEY RESOURCES TABLE
- EXPERIMENTAL MODEL AND STUDY PARTICIPANT DETAILS
  ○ Patients and samples

- CELL LINES
- METHOD DETAILS
  ○ E1/E2 antigen and ELISA

- NEUTRALISATION ASSAYS
- MUTANTS
- SEQUENCING
- QUANTIFICATION AND STATISTICAL ANALYSIS
  ○ Phylogenetics
  ○ Correlation between baseline binding and neutralisation
  ○ Association between non-viral factors and antibody response
  ○ Association between viral amino acids polymorphisms in E1/E2 and antibody response
  ○ Association between the N-linked glycosylation motifs and antibody response
  ○ Correlation between within patient viral nucleotide diversity and antibody response
  ○ Structural mapping and epitope localization of antibody-associated polymorphisms

## Star★Methods

### Key Resources Table

**Table:** 

REAGENT or RESOURCE	SOURCE	IDENTIFIER
Antibodies		
anti-human Ig HRP	Sigma	A0170; RRID: AB_257868
Biological samples		
Plasma samples from BOSON patients	BOSON Trial	NCT01962441
Chemicals, peptides, and recombinant proteins		
*Galanthus nivalis* agglutinin (GNA)	Sigma	L8275
PBS	Sigma	D8537
Tween 20	Sigma	P9416
BSA	Sigma	A9576
3,5,3′,5′-tetramethylbenzidine (TMB)	Sigma	ES022
Luciferase assay substrate	Promega	E1501
Critical commercial assays		
NEBNext Ultra Directional RNA Library Prep Kit	New England Biolabs	N\A
Illumina TruSeq or Ion Torrent Library (v1.0))	Illumina	N\A
Deposited data		
Boson HCV sequence data are deposited in GenBank under the following codes: KY620313-KY620880	GenBank	KY620313-KY620880
Experimental models: Cell lines		
HEK293T kidney embryo cell line	ECACC	12022001
HuH7 hepatoma cell	JRCB	JCRB0403
Oligonucleotides		
120 nt DNA oligonucleotide capture probes for HCV	IDT	Sequences available upon request form the lead author
Recombinant DNA		
Plasmid: UKN3A13.6	University of Nottingham	AY894683
phCMV5349 packaging vector	Ecole Normale Superieure de Lyon	N\A
pTG126 reporter plasmid	Ecole Normale Superieure de Lyon	N\A
Software and algorithms		
GraphPad Prism (version 10.4.1 (627))	Graphpad Inc	https://www.graphpad.com/
ChimeraX v1.7	University of California	https://www.cgl.ucsf.edu/chimerax/
AlphaFold 3	Google DeepMind	https://alphafoldserver.com/
ConSurf WEB SERVER	THE CONSURF SERVER	https://consurf.tau.ac.il/
R (version 4.1.1)	R Development Core Team	https://www.r-project.org/
NGPhylogeny server	LIRMM/ATGC	https://ngphylogeny.fr/
QUASR (v7.0120)	Bioconductor	https://www.bioconductor.org/packages/release/bioc/html/QuasR.html
CutAdapt (v1.7.1)	NBIS (National Bioinformatics Infrastructure Sweden).	https://cutadapt.readthedocs.io/en/stable/
Vicuna (v1.3)	Broad Institute	http://www.broadinstitute.org/scientific-community/science/projects/viral-genomics/viral-genomics-analysis-software
V-FAT (v1.0)	Broad Institute	https://www.broadinstitute.org/viral-genomics/v-fat

### Experimental Model And Study Participant Details

#### Patients and samples

The study was conducted in collaboration with STOP-HCV, a consortium sponsored by the Medical Research Council, United Kingdom, which contributed to study design. This study used samples from 60 patients with HCV infection selected from BOSON clinical trial^40^ (registration number: NCT01962441). Among these, 54 individuals were infected with HCV gt3a, two with gt3b, and one with gt2b. Further analysis in this study focused on samples from the 54 patients infected with gt3a viruses to control the impact of viral genetic heterogeneity. The gt3a patient cohort had a mean age of 52 years, comprising 13 females and 41 males. Reported ethnic backgrounds included White (*n* = 48), Asian (*n* = 4), Black (*n* = 1), and American Indian or Alaska Native (*n* = 1). ELISA based IgG binding and retroviral pseudotype-based neutralisation assays were implemented using samples recovered at baseline, without DAA treatment. The males were associated with lower antibody binding as reported in this study. All patients provided written informed consent before undertaking any study-related procedures. The study protocol was approved by each institution’s review board or ethics committee before study initiation.

### Cell Lines

HEK293T (ECACC 12022001) cells were used to express HCV E1/E2 glycoproteins for ELISA assays. Cell lysates were harvested 72 h post-transfection and used as antigen preparations. HuH7 hepatoma cells (a kind gift of Arvind Patel, MRC Center for Virus Research) were used as target cells in retroviral pseudotype-based neutralisation assays with murine leukemia virus cores bearing HCV E1/E2 glycoproteins. Cell lines were not authenticated in house. All cell lines were routinely tested for mycoplasma contamination using a BI EZ-PCR kit (Biological Industries 20-700-20) and found to be uninfected.

### Method Details

#### E1/E2 antigen and ELISA

The polyclonal antibody response directed to the HCV E1 and E2 envelope glycoproteins were detected using well characterized antigen representing genotype 3a (UKN3A13.6,^41^ also coded as UKNP3.2.1 in some studies when included in panels of patient-isolated clones, essentially as described previously^18^). Briefly, wells of Maxisorp 96-well plates were coated with *Galanthus nivalis* agglutinin (GNA) (Sigma; 1μg⋅mL^–1^) and blocked with PBS containing 5% BSA. Cell lysates of HEK293T cells transfected with constructs expressing the genes encoding these E1/E2 proteins were recovered 72 h after transfection and diluted 1:5 in PBS containing 0.05% Tween (PBST) before adding to coated wells. After washing, patient plasma samples were added, diluted in PBST-5% BSA, between 1:100 and 1:10000 to titrate seroreactivity. A dilution of 1:300 was subsequently selected for further experimentation. Bound antibody was detected using anti-human Ig HRP and 3,5,3′,5′-tetramethylbenzidine (TMB) substrate was used to detect bound antibodies and the reaction was stopped after 15 min. The ELISA experiments were performed in duplicate and for analysis we used the mean for each sample.

### Neutralisation Assays

Murine Leukemia Virus cores pseudotyped with the HCV E1/E2 genes representing isolate UKN3A13.6 were created as previously described,^68^ using a luciferase reporter construct.^69^ Virus pseudotypes were incubated with sera (heat-inactivated prior to use at 56°C for 30 min) for one hour at 25°C before adding to target HuH7 cells for four hours at 37°C, in a 5% CO_2_ incubator. Following infection, media was exchanged, and cells incubated for a further 72 h. Following cell lysis, infection was assayed using a Promega luciferase assay substrate. Luciferase activity was measured, and data were normalized to an uninhibited control and a preparation of pseudotypes created without a viral glycoprotein. Percentage values were calculated by normalizing to an uninhibited infection (100%) and the signal generated in an assay performed with pseudotypes lacking viral glycoprotein (0%). The neutralisation experiments were performed in triplicate and for analysis we used the mean for each sample.

### Mutants

Single point mutants were generated using the wild-type UKN3A13.6 plasmid (accession AY894683.1) as template. Mutagenesis was performed using a Q5 Mutagenesis Kit (NEB) following the manufacturer’s protocol (primers available on request). Mutants T501N and E533K were generated, and infectivity assessed using the pseudotype model described above.

### Sequencing

Sequencing was performed as described previously.^21^ Briefly, viral RNA was isolated from 500 μL plasma using the NucliSENS magnetic extraction system (bioMerieux). Libraries were prepared using the NEBNext Ultra Directional RNA Library Prep Kit for Illumina (New England BioLabs) with a maximum of 10 ng total RNA template. 500 ng of pooled library was enriched using the xGen Lockdown protocol from Integrated DNA Technologies (IDT) (Rapid Protocol for DNA Probe Hybridization and Target Capture Using an Illumina TruSeq or Ion Torrent Library (v1.0)) with equimolar-pooled 120-nt DNA oligonucleotide probes (IDT) followed by a 12-cycle, modified, on-bead, post-enrichment PCR re-amplification step. The cleaned post-enrichment library was normalized with the aid of qPCR and sequenced with 151-base paired-end reads on a single run of the Illumina MiSeq using v2 chemistry. De-multiplexed sequence-read pairs were trimmed of low-quality bases using QUASR (v7.0120) and of adaptor sequences using CutAdapt (v1.7.1). The remaining read pool was screened against a BLASTn database containing 165 HCV genomes, which covered its diversity both to choose an appropriate reference and to select those reads that formed a population for *de novo* assembly with Vicuna (v1.3). The assembly was finished with V-FAT v1.0 (http://www.broadinstitute.org/scientific-community/science/projects/viral-genomics/v-fat).

### Quantification and Statistical Analysis

#### Phylogenetics

Whole-genome viral nucleotide consensus sequences for each patient in the study (*n* = 57) and gt3a HCV reference sequences (*n* = 9) were aligned using MAFFT^70^ with default settings. The alignment file containing only the E1 and E2 regions was uploaded to the NGPhylogeny server (https://ngphylogeny.fr/) and a maximum-likelihood tree was generated with default settings. We used R Statistical Software (Version 4.1.1) and “ape” and “phytools” packages^71,72^ to read the newick format tree and root the tree at midpoint and plot it. We used linear regression in R (lm function) to test the impact of number of amino acid differences between the UKN3A13.6 reference and the viral consensus sequences for each patient.

#### Correlation between baseline binding and neutralisation

To test for the relationship between baseline binding and neutralisation, we used univariable linear regression model, calculating the *p* value and R squared. Binding was the explanatory variable and neutralisation was the response variable in the model. The 95% confidence interval for the best-fit line was calculated by “predict” function from “stats” package in R.

#### Association between non-viral factors and antibody response

A multivariable linear regression model was used to test for the association between clinical phenotypes and antibody response. Antibody response was used as the outcome variable and the patient’s sex, cirrhosis status, prior IFN-based treatment status, age and log of baseline viral load were included as covariates in the model. The *p* values for the associations were from linear regression model. The “metafor” package in R was used to plot the *p* values and 95% confidence intervals of the association test as a forest plot. To test the association between three *IFNL4* gene haplotypes and antibody response, the univariable linear regression was performed. The antibody response was the explanatory variable and the *IFNL4* gene haplotypes were the response variable in the model. To test for HLA alleles association with antibody response, we used antibody response as the explanatory variable and HLA alleles as response variables to perform univariable linear regressions. The HLA allele data were imputed as previously described.^73^ The multiple tests were corrected at a false-discovery rate (FDR) of 20%. All the *p* values were from the linear regression models.

#### Association between viral amino acids polymorphisms in E1/E2 and antibody response

We used linear regression to test for association between the presence and absence of each amino residue at variable sites E1 and E2 and antibody response, including patient’s sex, cirrhosis status, prior IFN-based treatment status, log of baseline viral load and *IFNL4* gene haplotypes as covariates. The threshold number for the tested residues was set to greater than ten isolates. To balance discovery with error control, we used both 5% and 20% False-discovery rate to account for multiple testing. The associated viral amino acids sites calculated from the tests were performed linear regression for association with antibody response, including the same confounding factors above. All the *p* values were from the linear regression models.

#### Association between the N-linked glycosylation motifs and antibody response

A linear regression model was used to test for association between the presence or absence of each N-linked glycosylation motif at glycosylated sites at E1 and E2 proteins. The multivariate model included sex, cirrhosis status, prior IFN-based treatment status, log of baseline viral load and *IFNL4* gene haplotypes as covariates to control for possible confounding. We only tested sites where there was some variability in the motifs with a threshold count of 10 or more. False-discovery rate (FDR) of 20% was used to correct for multiple testing where we combined the *p* values generated from binding and neutralisation association tests in one FDR procedure.

#### Correlation between within patient viral nucleotide diversity and antibody response

Univariable linear regression model was used to test for association between within patient viral diversity at the nucleotide level and antibody response. The mean nucleotide diversity was calculated from next-generation sequencing reads presenting as the measure of within patient viral nucleotide diversity. The mean nucleotide diversity across the whole genome, E1 combined with E2, E1, E2 and hypervariable region 1 were tested for the correlation with antibody response. All the *p* values were from the linear regression models.

#### Structural mapping and epitope localization of antibody-associated polymorphisms

To investigate the structural and evolutionary context of polymorphisms associated with antibody responses, we mapped identified amino acid variants onto the high-resolution crystal structure of the HCV genotype 3a E1/E2 heterodimer (PDB ID: 8RJJ^12^). A multiple sequence alignment of E1/E2 sequences from our cohort was generated and used as input for the ConSurf web server (https://consurf.tau.ac.il/), which computes residue-specific evolutionary conservation scores. These scores were then projected onto the E1/E2 structure to visualize conserved versus variable regions. Structural visualisation, highlighting of polymorphic residues, and overlay of antibody epitope footprints were carried out using UCSF ChimeraX (version 1.9).^74^ Mutant models incorporating the observed polymorphisms (T234K, T476S, S501N, E533K, D653N) were generated using AlphaFold3^75^ to assess their potential structural impact relative to the consensus E1/E2 sequence. All figures were prepared in ChimeraX.

## Supplementary Material

Supplemental information can be found online at https://doi.org/10.1016/j.celrep.2025.116418.

Supplementary Material

## Data Availability

The HCV sequence data used in this study come from the BOSON clinical trial (ClinicalTrials.gov identifier: NCT01962441). BOSON HCV sequence data have been deposited in GenBank under the following codes GenBank: KY620313–KY620880, and are publicly available. The rest of the data reported in this paper will be shared by the lead contact upon request. This paper does not report original code. Any additional information required to reanalyze the data reported in this paper is available from the lead author upon request.

## In brief

Wang et al. investigate why antibody responses to the hepatitis C virus vary between individuals. They find that specific genetic changes in both the virus (genotype 3a) and the infected person significantly impact antibody effectiveness. Understanding this host-virus interplay is crucial for designing vaccines against diverse HCV strains.

## Highlights

- Genetic variation in host *IFNL4* gene influences anti-HCV antibody binding
- Naturally occurring amino acid variation in HCV E2 modulates antibody responses
- N-glycosylation motif variants in E1/E2 proteins affect antibody responses
- Intra-patient HCV HVR1 diversity correlates with antibody binding strength
