# Supplementary material for: Interplay of host and viral genetic variations in modulating antibody responses to genotype 3a hepatitis C virus: Implications for vaccine design
Source: Cell Rep. Author manuscript; Available in PMC 2026 Jan 20. (PMC7618651; doi:10.1016/j.celrep.2025.116418)
Supplement: Supplementary Material [file EMS212071-supplement-Supplementary_Material.zip › 1-s2.0-S2211124725011891-mmc1.pdf]

Cell Reports, Volume *44*

Supplemental information

Interplay of host and viral genetic variations in modulating antibody responses to genotype 3a hepatitis C virus: Implications for vaccine design

Zhiqing Wang, Isla Humphreys, Jocelyn Quistrebert, Haiting Chai, Robert Stass, Josh Dhir, Alexandru Nisioi, Paul Radford, STOP-HCV consortium, Jonathan K. Ball, William L. Irving, Thomas A. Bowden, Paul Klenerman, Eleanor Barnes, Jane A. McKeating, Alexander W. Tarr, and M. Azim Ansari

Genotype-3 Baseline

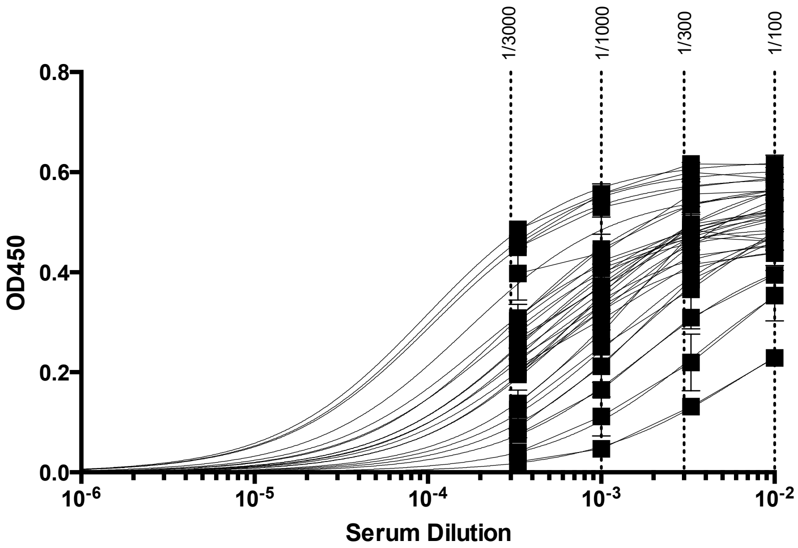

Figure S1. Titration of antibody reactivity to UKN3A13.6 E1/E2 proteins in ELISA.
E1/E2 proteins were expressed in HEK293T cells and antibody binding assessed using ELISA. The signal in these assays is corrected for background reactivity binding to a control cell lysate derived from mock transfected HEK293T cells. The values on the x-axis represent the dilution factor of each serum sample.

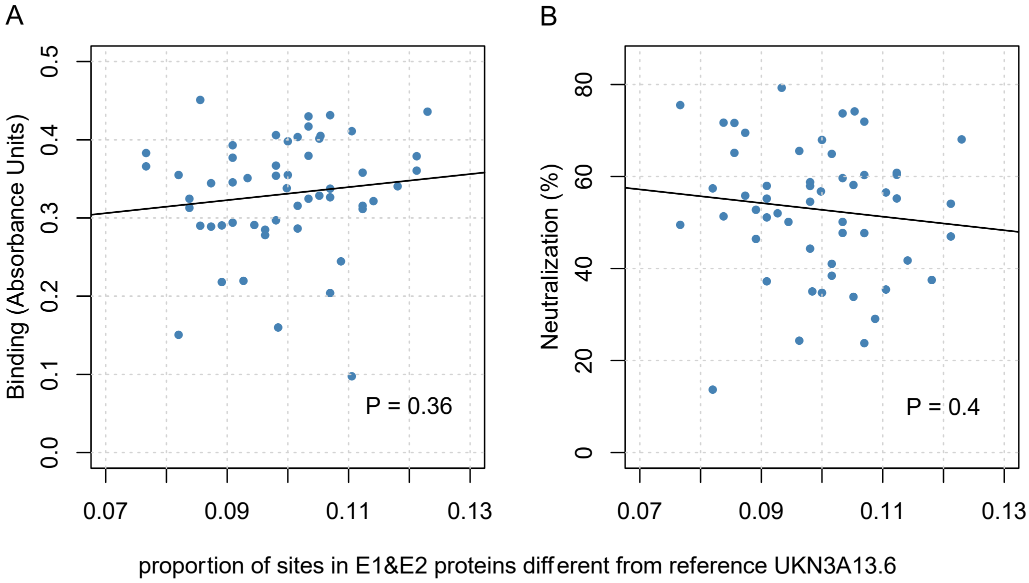

Figure S2. Correlation between antibody response and E1/E2 amino acid divergence from the reference antigen (UKN3A13.6).

(A) Binding.

(B) Neutralization.

Linear regression p-values for the association are shown. The x-axis indicates the proportion of amino acid differences between study isolates and the reference UKN3A13.6 antigen. Each blue dot indicates a study sample and the solid black lines show the best fit linear regression lines and grey area indicate its 95% confidence interval. *P* values are estimated using a linear regression model.

A

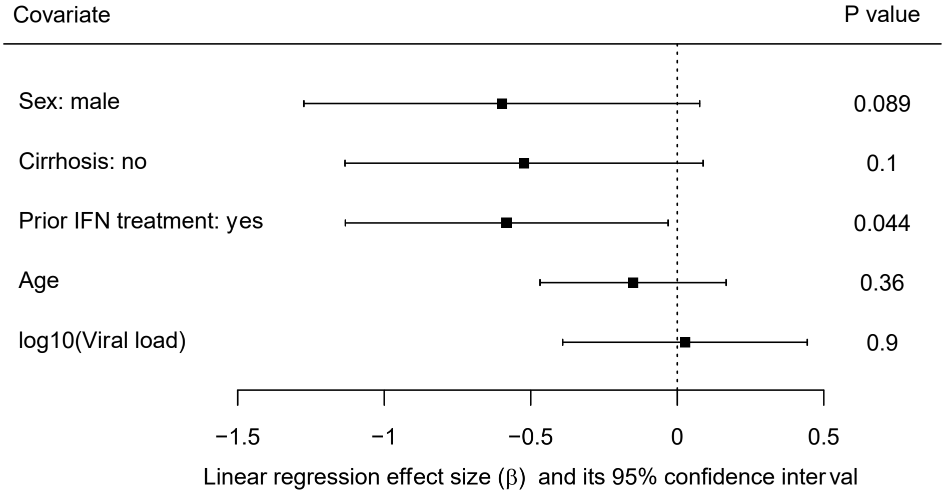

B

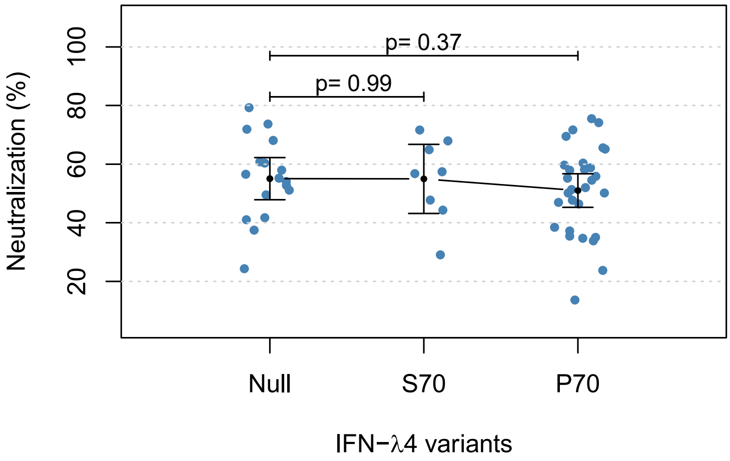

Figure S3. The impact of host genetic and non-genetic factors on antibody neutralization response in HCV infection.

(A) Forest plot of the effect sizes and their confidence intervals for non-viral factors tested against neutralization. The squares show the linear regression estimated effect sizes for each covariate and the lines show its 95% confidence interval. The *p* values (from linear regression) for each covariate is shown on the right (*n* = 54).

(B) Neutralization stratified by the host IFNλ4 protein haplotypes. The black dots and lines indicate the mean and 95% confidence interval (CI) for each group. *P* values were calculated using linear regression model.

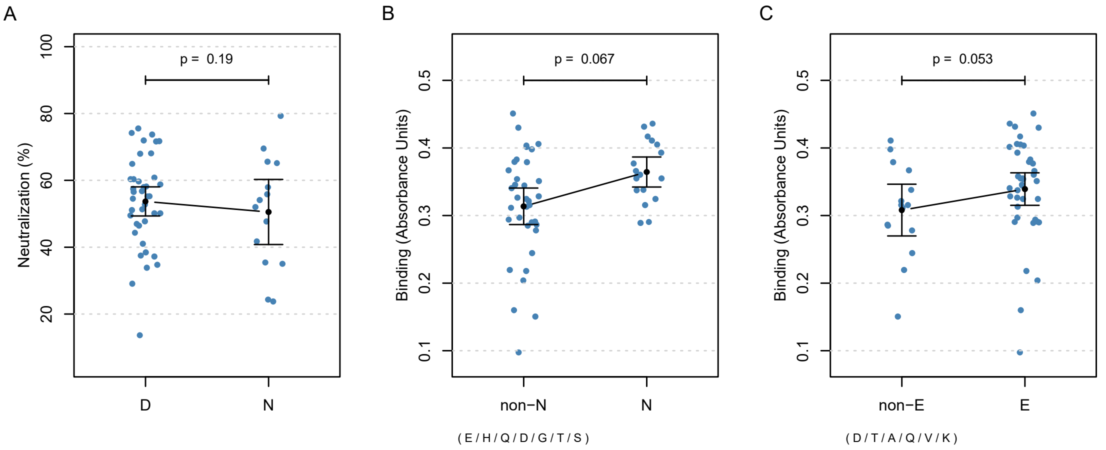

Figure S4. Association between HCV amino acid polymorphisms in E1 and E2 proteins and antibody response.
(A) Association between site 653 and neutralization.
(B) Association between site 501 and binding.
(C) Association between site 533 and binding.
The black dots and lines indicate the mean and 95% confidence interval (CI) for each group. *P* values were calculated using linear regression model.

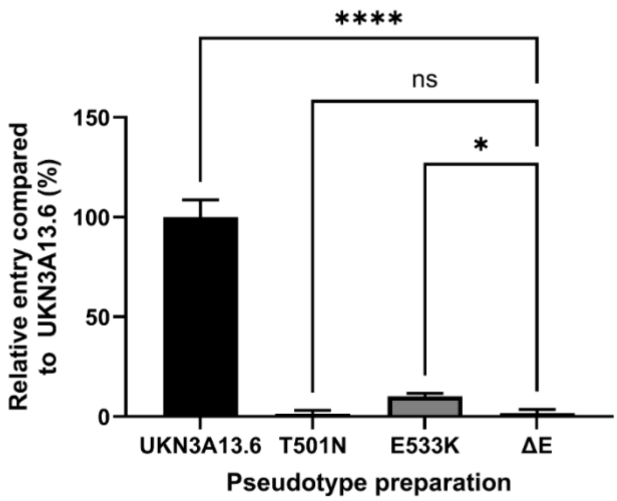

Figure S5. Infectivity of HCV mutants using E1/E2 Pseudo-particles.

Infection was performed using HuH7 cells, infected with pseudotypes bearing the wild-type UKN3A13.6 variant glycoprotein, or single aa mutants T501N or E533K. A preparation possessing pseudotypes created in the absence of E1/E2 (ΔE) was used as a negative control. Statistical comparisons were performed using one-way ANOVA with Dunnett's correction for multiple comparisons. **** $p < 0.0001$, * $p < 0.05$, n.s. not significant.

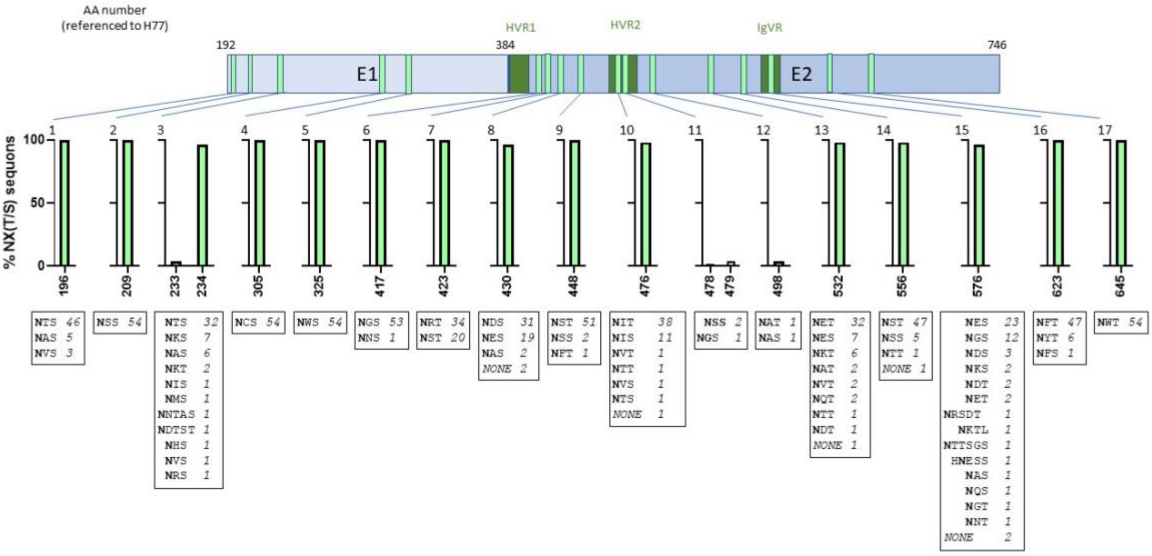

Figure S6. Locations and amino acid sequences of glycosylation sequons in the E1/E2 proteins in HCV genotype 3a samples in the study sequences.

Illustrated with a graphical representation of the E1 and E2 genes, the proportion of sequences that possessed N-linked glycosylation sequons (NX(T/S)) at each of the sites is indicated. Amino acid sequences of the glycosylation sequons and their frequency are listed, as represented in the dataset. The locations of the hypervariable region 1 (HVR1), hypervariable region 2 (HVR2) and Intergenotypic variable region (IgVR) are highlighted, as well as the amino acid numbers of the start of E1, the E1/E2 boundary, and the end of E2 (referenced to strain H77, Genbank accession number AAB67036.1)

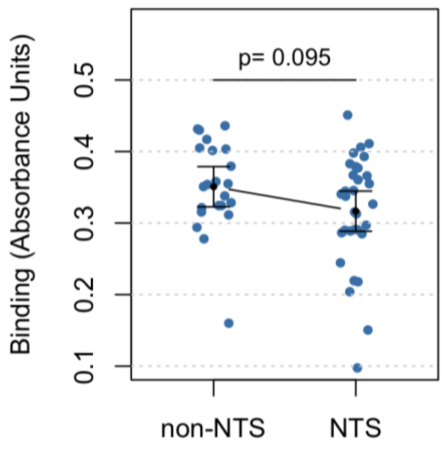

Figure S7. Association between glycosylation site N234 and binding.
The black dots and lines indicate the mean and 95% confidence interval (CI) for each group. *P* values were calculated using linear regression model. The non-NTS group comprised of NKS (*n* = 7), NAS (*n* = 6), NKT (*n* = 2), NHS, NIS, NMS, NVS and NMS polymorphisms at this glycosylation site.

A

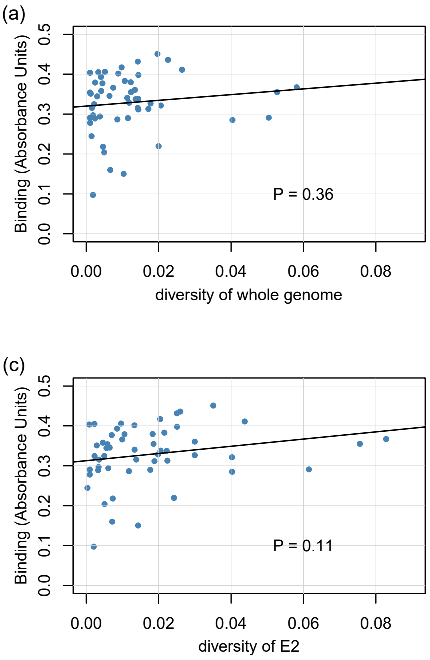

B

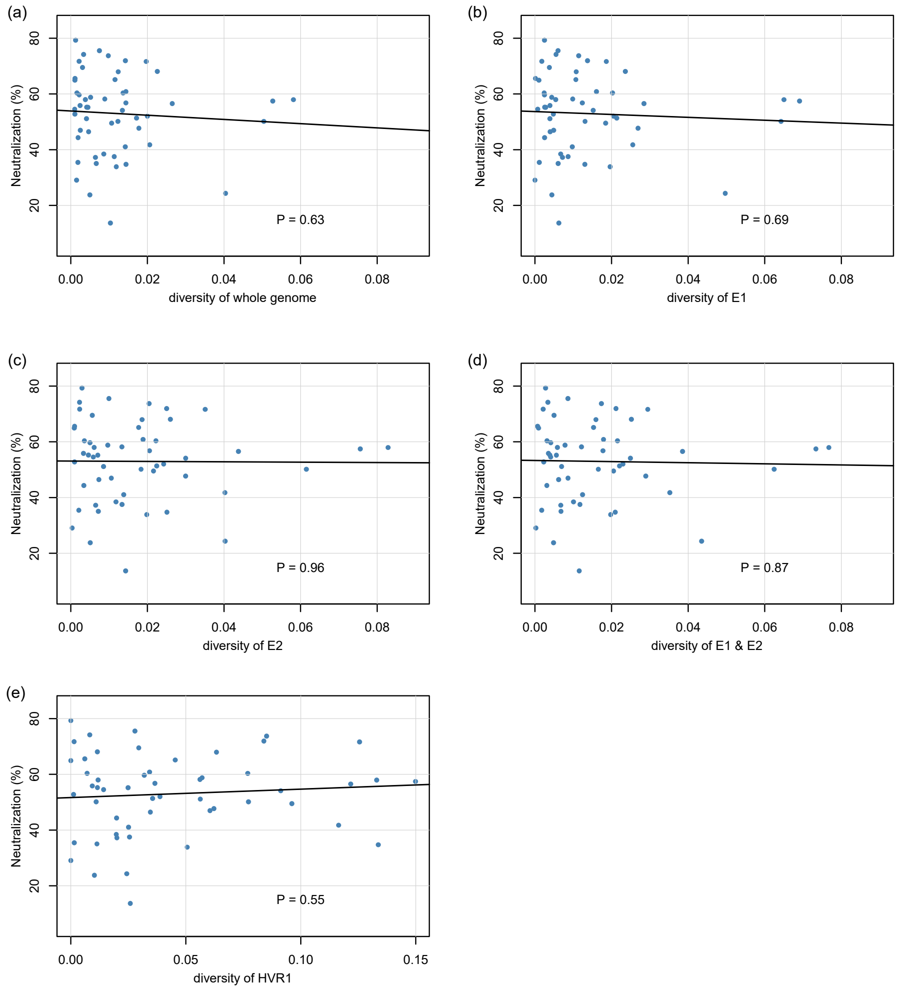

Figure S8. Correlates of intra-patient viral nucleotide diversity and antibody responses.
(A) Correlates of binding and intra-patient viral nucleotide diversity.

The intra-patient viral diversity in whole viral sequences (a), E1 protein region (b), E2 protein region (c), E1 and E2 region together (d) region.
(B) Correlates of neutralization and intra-patient viral nucleotide diversity.
The intra-patient viral diversity in whole viral sequences (a), E1 protein region (b), E2 protein region (c), E1 and E2 region together (d) and HVR1 (e) region.
The solid black lines show the best fit linear regression line and grey area indicates its 95% confidence interval. *P* values for the slopes are from the linear regression model.

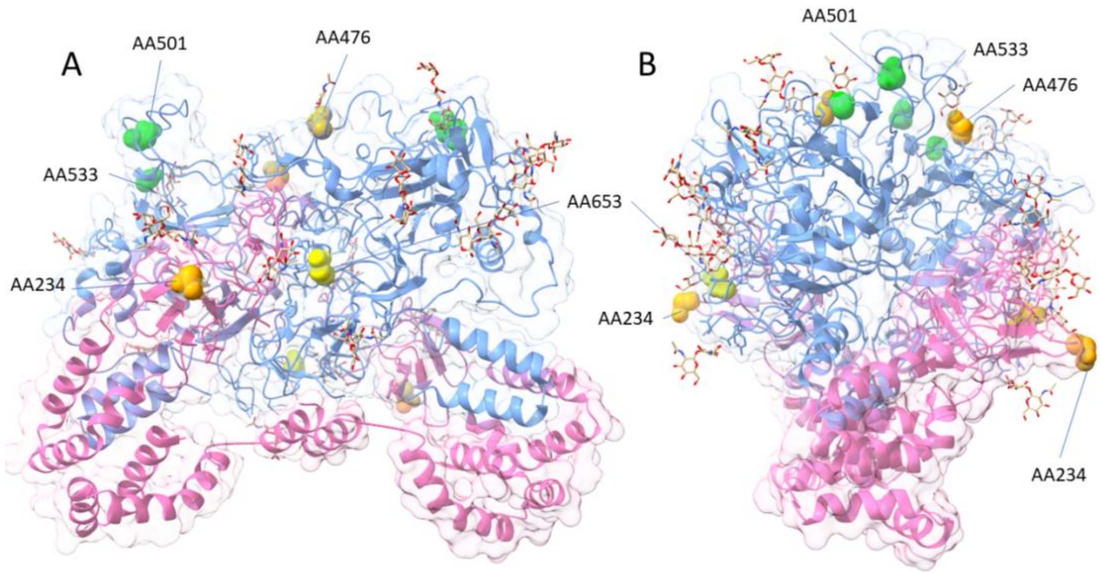

Figure S9. Location of sites associated with antibody neutralization/binding phenotype in a structure of the E1/E2 heterocomplex.

In this structure of E1/E2 (PDB 8RJJ), E1 is highlighted in pink, with E2 highlighted in blue. Carbohydrates associated with these proteins are presented as wireframes, with a space-filling representation of the amino acid side chains representing the two amino acids associated with neutralization phenotype (AA501 and AA533, green), and the amino acid associated with antibody binding (AA653, yellow). Asparagine residues 234 and 476 modified with *N*-linked glycans are highlighted in orange. The left figure is a 'side-on' presentation of the 'dimer of heterodimer' structure. The right one is for the structure rotated through 90° in a vertical axis.

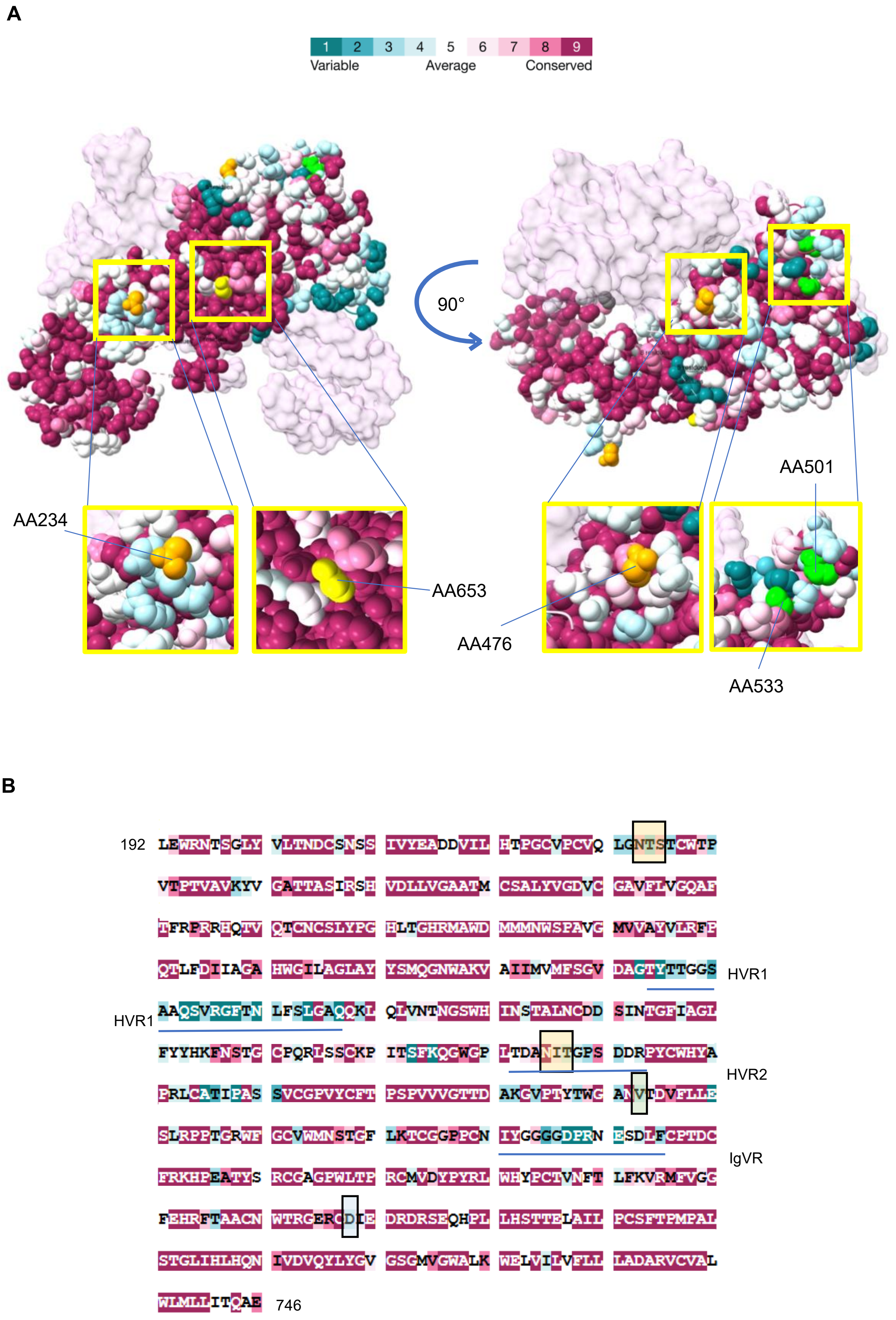
A
1 2 3 4 5 6 7 8 9
Variable
Average
Conserved
90°
AA234
AA653
AA476
AA501
AA533
B
192 LEWRNTSGLY VLTNDCSNSS IVYEADDVIL HTPGCVPCVQ LGNTSTCWTP
VTPTVAVKYV GATTASIRSH VDLLVGAATM CSALYVGDVC GAVFLVGQAF
TFRPRRHQTV QTCNCSLYPG HLTGHRMAWD MMMNWSPAVG MVVAYVLRFP
QTLFDIIAGA HWGILAGLAY YSMQGNWAKV AIIMVMFSGV DAGTYTTGGS HVR1
HVR1 AAQSVRGFTN LFSLGAQQKL QLVNTNGSWH INSTALNCDD SINTGFIAGL
FYYHKFNSTG CPQRLSSCKP ITSFKQGWGP LTDANITGPS DDRPYCWHYA
HVR2
PRLCATIPAS SVCGPVYCFT PSPVVVGTTD AKGVPTYTWG ANVTDVFLLE
SLRPPTGRWF GCVWMNSTGF LKTCGGPPCN IYGGGDPRN ESDLFCPTDC
IgVR
FRKHPEATYS RCGAGPWLTP RCMVDYPYRL WHYPCTVNFT LFKVRMFVGG
FEHRFTAACN WTRGERCDIE DRDRSEQHPL LHSTTELAIL PCSFTPMPAL
STGLIHLHQN IVDVQYLYGV GSGMVGWALK WELVILVFLL LADARVCVAL
WLMLLITQAE 746

Figure S10. Structural and sequence context of antibody-associated amino acid polymorphisms in HCV E1/E2.

(A) Structural mapping of antibody-associated polymorphisms onto the E1/E2 heterodimer (PDB: 8RJJ), with ConSurf analysis used to visualise site-specific amino acid variability. One copy of the E1/E2 heterodimer is shown with a space-filling representation of side chains coloured by evolutionary conservation. Residues associated with neutralisation sensitivity (501 and 533) are highlighted in green; the residue associated with antibody binding (653) is shown in yellow. N-linked glycosylated asparagine residues at positions 234 and 476 are shown in orange. The second E1/E2 heterodimer is rendered as a surface to illustrate the quaternary interface. The left panel presents a side view of the E1/E2 dimer-of-heterodimers; the right panel shows the same structure rotated 90° along the horizontal axis. (B) Linear sequence representation of E1 and E2 proteins, highlighting regions of sequence conservation and variability. Hypervariable regions (HVR1, HVR2), the inter-genotypic variable region (IgVR), and antibody-associated polymorphic sites are annotated for reference.

HLA allele	Allele count	P value	Effect size	Standard error	q value
DPA1*02:01	16	0.068	0.493	0.265	0.409
DQB1*03:01	15	0.176	-0.344	0.251	0.409
B*07:02	12	0.195	-0.344	0.262	0.409
C*06:02	10	0.205	-0.389	0.303	0.409
DRB3*01:01	20	0.216	-0.351	0.280	0.409
C*07:02	14	0.219	-0.317	0.255	0.409
DRB3*03:01	12	0.237	0.314	0.263	0.409
DQA1*05:01	21	0.321	-0.241	0.241	0.409
DPA1*01:03	51	0.363	-0.209	0.228	0.409
C*07:01	15	0.371	0.228	0.253	0.409
DQB1*02:01	10	0.378	0.312	0.351	0.409
DRB1*03:01	10	0.378	0.312	0.351	0.409
A*03:01	15	0.391	-0.219	0.253	0.409
A*01:01	14	0.533	0.161	0.257	0.409
DRB3*99:01	28	0.537	0.122	0.196	0.409
A*24:02	10	0.550	0.212	0.352	0.409
A*02:01	21	0.569	-0.139	0.243	0.409
DRB4*99:01	49	0.574	-0.118	0.208	0.409
DRB4*01:01	25	0.574	0.118	0.208	0.409
DRB3*02:02	26	0.621	-0.094	0.188	0.409
DQA1*01:01	15	0.627	-0.135	0.277	0.409
DQA1*03:01	12	0.688	0.118	0.293	0.409
C*04:01	12	0.700	-0.103	0.266	0.409
DQB1*03:02	10	0.701	0.136	0.353	0.409
DRB5*99:01	53	0.787	0.073	0.270	0.409
DQB1*06:02	17	0.787	-0.073	0.270	0.409
DRB1*15:01	17	0.787	-0.073	0.270	0.409
DRB5*01:01	17	0.787	-0.073	0.270	0.409
DQA1*02:01	18	0.806	0.066	0.267	0.409
DRB1*07:01	18	0.806	0.066	0.267	0.409
DQA1*01:02	21	0.827	0.051	0.229	0.409
B*08:01	12	0.841	0.059	0.293	0.409
DQB1*02:02	16	0.992	0.003	0.273	0.467

Table S1. The association between HLA alleles and binding.
The tested HLA alleles (count >= 10) are listed in the table. The *p* values, effect size and standard error are from linear regression tests and are shown in the table. Q value was estimated using qvalue function in R.

HLA allele	Allele count	P value	Effect size	Standard error	q value
A*03:01	15	0.022	-0.571	0.243	0.742
DRB3*02:02	26	0.128	-0.285	0.185	0.837
DRB3*03:01	12	0.138	0.392	0.261	0.837
A*01:01	14	0.144	0.375	0.253	0.837
C*07:01	15	0.146	0.369	0.250	0.837
DQA1*05:01	21	0.195	-0.315	0.239	0.837
C*07:02	14	0.215	-0.319	0.255	0.837
B*07:02	12	0.276	-0.290	0.263	0.837
A*24:02	10	0.311	0.358	0.350	0.837
DQB1*03:01	15	0.341	-0.243	0.253	0.837
A*02:01	21	0.342	0.232	0.241	0.837
B*08:01	12	0.383	0.256	0.291	0.837
DQA1*01:01	15	0.450	-0.210	0.276	0.837
C*06:02	10	0.476	-0.220	0.306	0.837
DRB4*01:01	25	0.530	0.132	0.208	0.837
DRB4*99:01	49	0.530	-0.132	0.208	0.837
DRB3*99:01	28	0.534	0.123	0.196	0.837
DPA1*02:01	16	0.548	0.165	0.272	0.837
C*04:01	12	0.553	0.159	0.265	0.837
DQA1*02:01	18	0.591	0.144	0.266	0.837
DRB1*07:01	18	0.591	0.144	0.266	0.837
DQB1*03:02	10	0.630	0.171	0.353	0.837
DRB3*01:01	20	0.634	-0.136	0.284	0.837
DQA1*01:02	21	0.669	0.098	0.229	0.837
DQA1*03:01	12	0.738	0.098	0.293	0.837
DRB5*99:01	53	0.739	0.090	0.270	0.837
DQB1*06:02	17	0.739	-0.090	0.270	0.837
DRB1*15:01	17	0.739	-0.090	0.270	0.837
DRB5*01:01	17	0.739	-0.090	0.270	0.837
DQB1*02:02	16	0.761	0.084	0.273	0.837
DPA1*01:03	51	0.895	-0.030	0.230	0.897
DQB1*02:01	10	0.897	0.046	0.354	0.897
DRB1*03:01	10	0.897	0.046	0.354	0.897

Table S2. The association between HLA alleles and neutralization.
The tested HLA alleles (count >= 10) are listed in the table. The *p* values, effect size and standard error are from linear regression and are shown in the table. Q value was estimated using qvalue function in R.

The tested site	The most associated amino acid	Amino acids in reducing frequency order at the tested site	P value	Effect size	Standard error	q value
**653**	**D**	**DN**	**6.80E-05**	**1.133**	**0.259**	**0.006**
407	A	APS	0.009	-0.698	0.254	0.372
466	R	KRNESG	0.025	0.571	0.246	0.372
500	S	SALRTKV	0.036	-0.536	0.248	0.372
540	E	EKTQNR	0.038	-0.553	0.259	0.372
501	S	SNTDGEHQ	0.041	-0.556	0.264	0.372
388	T	TISV	0.043	-0.595	0.285	0.372
235	T	TKAHIMRV	0.048	-0.521	0.256	0.372
561	V	VLTI	0.05	0.489	0.242	0.372
387	I	TVIL	0.058	0.611	0.315	0.372
280	M	MVLI	0.068	-0.553	0.297	0.372
396	T	ATVILP	0.078	0.611	0.339	0.372
392	A	AVPTMQEI	0.085	-0.447	0.254	0.372
386	R	YRHT	0.093	0.446	0.26	0.372
446	K	KRSQ	0.096	-0.468	0.276	0.372
401	S	SGKNTAQR	0.098	-0.421	0.249	0.372
574	E	GEDRAKPST	0.106	-0.483	0.293	0.372
384	S	SETNQDGAHRY	0.108	0.497	0.303	0.372
575	G	GEKRMQS	0.11	0.455	0.279	0.372
576	N	NDSGEKTACPV	0.114	0.475	0.294	0.372
533	E	EKAQVDT	0.116	0.457	0.285	0.372
495	D	DGESTKNAQR	0.121	0.497	0.315	0.372
578	D	DGHNRCPS	0.122	0.473	0.301	0.372
408	Q	QKRNS	0.124	-0.414	0.264	0.372
478	S	TSN	0.132	-0.455	0.297	0.372
576b	R	RKSNGQDHTYEP	0.133	-0.523	0.342	0.372
553	T	TAVS	0.14	0.377	0.251	0.372
404	S	SNTAQKHR	0.154	-0.373	0.258	0.387
395	G	GSTANDQFHK	0.161	-0.394	0.276	0.387
531	A	EAGTM	0.163	-0.38	0.268	0.387
497	V	VI	0.168	-0.419	0.299	0.387
481	D	DENAGH	0.177	-0.373	0.272	0.387
241	P	PSA	0.184	0.349	0.258	0.387
237	K	TKMES	0.188	0.451	0.338	0.387
438	I	ILMV	0.191	-0.391	0.295	0.387
375	M	MIVL	0.194	-0.358	0.272	0.387
391	A	SATNLQR	0.235	-0.391	0.325	0.437
410	N	NKRHPS	0.24	-0.308	0.258	0.437
400	A	ATVSYGKL	0.27	0.28	0.251	0.466
202	V	VI	0.288	-0.282	0.263	0.466
641	D	TDSENA	0.289	0.344	0.321	0.466
398	G	GSTRVFIKM	0.297	-0.261	0.247	0.466
232	D	DNTHAEQS	0.297	-0.278	0.264	0.466

The tested site	The most associated amino acid	Amino acids in reducing frequency order at the tested site	P value	Effect size	Standard error	q value
405	P	PLQRVMAKSTW	0.302	-0.277	0.266	0.466
394	R	RHQYSFGKV	0.315	-0.27	0.266	0.478
397	S	SRHNFLQYGKAEMW	0.323	0.268	0.268	0.481
464	F	FSHAYN	0.404	0.215	0.255	0.544
249	R	KRE	0.409	-0.214	0.256	0.544
522	K	KREGMQ	0.431	0.209	0.263	0.549
576a	P	PHRLSTFAENV	0.443	-0.205	0.265	0.549
524	V	AVTM	0.48	0.189	0.265	0.578
546	S	SNGRAKQ	0.537	0.169	0.272	0.609
498	P	PQSLNAKR	0.538	0.173	0.279	0.609
490	A	AP	0.54	-0.186	0.301	0.609
521	A	ARVDEISLT	0.572	-0.156	0.273	0.616
576d	E	EGDKTANQS	0.582	0.143	0.259	0.616
608	M	MLI	0.599	0.165	0.311	0.627
471	S	PST	0.643	-0.137	0.294	0.645
399	L	FLISV	0.655	0.128	0.286	0.645
337	V	VIL	0.683	0.131	0.319	0.645
223	A	TAI	0.686	-0.129	0.317	0.645
591	E	EDGAK	0.694	0.12	0.302	0.645
492	R	RK	0.716	-0.107	0.294	0.645
496	V	TIVDELNS	0.717	0.118	0.323	0.645
442	F	FIVL	0.739	0.106	0.316	0.645
208	S	SP	0.752	0.102	0.321	0.645
414	V	VI	0.764	-0.078	0.258	0.645
431	D	DEA	0.775	-0.076	0.265	0.645
227	I	IV	0.785	0.076	0.278	0.645
479a	S	PSDT	0.813	-0.066	0.277	0.661
580	F	FLIHVAMSTY	0.831	0.059	0.275	0.663
314	S	ST	0.861	-0.048	0.274	0.663
454	Q	QEHRDGLY	0.892	-0.044	0.323	0.681
483	K	KR	0.942	0.02	0.269	0.705
424	R	RS	0.959	-0.014	0.272	0.705
528	T	TNSDGQ	0.977	0.009	0.302	0.705
376	V	VI	0.985	-0.006	0.315	0.705

Table S3. Statistical analysis of the association between E1/E2 amino acid variations and antibody binding.

The table presents results from linear regression models testing the association between antibody binding and polymorphisms at 77 sites (in total 123 residues were tested at these 77 sites, but only the most associated residue per site is shown in the table, numbered according to the H77 reference sequence). P values, effect sizes, and standard errors are reported, along with q-values representing FDR-corrected p-values to account for multiple testing. Associations at 20% FDR are highlighted in bold.

The tested site	The most associated amino acid	Amino acids in reducing frequency order at the tested site	P value	Effect size	Standard error	q value
**501**	**N**	**SNTDGEHQ**	**0.001**	**0.963**	**0.274**	**0.108**
**533**	**E**	**EKAQVDT**	**0.003**	**0.909**	**0.295**	**0.187**
561	V	VLTI	0.024	0.616	0.264	0.403
235	T	TKAHIMRV	0.025	-0.649	0.279	0.403
471	P	PST	0.025	0.691	0.298	0.403
540	E	EKTQNR	0.029	-0.644	0.285	0.403
496	V	TIVDELNS	0.029	0.765	0.34	0.403
478	S	TSN	0.038	-0.686	0.321	0.403
376	V	VI	0.039	0.708	0.333	0.403
396	V	ATVILP	0.039	-0.785	0.37	0.403
466	K	KRNESG	0.044	-0.545	0.263	0.403
384	S	SETNQDGAHRY	0.045	0.678	0.33	0.403
227	I	IV	0.089	-0.516	0.298	0.568
410	K	NKRHPS	0.093	0.526	0.306	0.568
404	S	SNTAQKHR	0.099	-0.475	0.283	0.568
531	E	EAGTM	0.121	0.442	0.28	0.618
392	A	AVPTMQEI	0.122	-0.444	0.282	0.618
398	G	GSTRVFIKM	0.125	-0.42	0.269	0.618
521	A	ARVDEISLT	0.134	-0.452	0.296	0.63
490	A	AP	0.154	-0.474	0.327	0.663
576a	P	PHRLSTFAENV	0.172	0.393	0.283	0.663
495	D	DGESTKNAQR	0.181	0.476	0.35	0.663
546	S	SNGRAKQ	0.187	0.397	0.296	0.663
653	D	DN	0.189	0.445	0.334	0.663
394	H	RHQYSFGKV	0.202	-0.45	0.347	0.663
401	G	SGKNTAQR	0.204	-0.406	0.315	0.663
314	S	ST	0.205	0.383	0.298	0.663
479a	S	PSDT	0.208	-0.385	0.301	0.663
500	S	SALRTKV	0.236	-0.34	0.283	0.693
446	K	KRSQ	0.242	-0.366	0.309	0.693
524	V	AVTM	0.247	0.341	0.29	0.693
576d	G	EGDKTANQS	0.251	0.366	0.314	0.693
414	V	VI	0.276	0.31	0.282	0.693
407	P	APS	0.281	-0.344	0.315	0.693
498	P	PQSLNAKR	0.294	0.325	0.306	0.693
424	R	RS	0.306	-0.308	0.297	0.693

The tested site	The most associated amino acid	Amino acids in reducing frequency order at the tested site	P value	Effect size	Standard error	q value
397	S	SRHNFLQYGKAEMW	0.307	-0.306	0.296	0.693
280	M	MVLI	0.315	-0.341	0.336	0.693
400	A	ATVSYGKL	0.324	-0.278	0.278	0.693
386	Y	YRHT	0.325	0.276	0.277	0.693
553	T	TAVS	0.328	0.277	0.281	0.693
608	L	MLI	0.339	-0.349	0.361	0.693
241	S	PSA	0.356	-0.298	0.319	0.693
249	K	KRE	0.366	-0.263	0.288	0.696
375	M	MIVL	0.373	0.273	0.303	0.697
388	T	TISV	0.386	-0.286	0.327	0.698
408	K	QKRNS	0.457	-0.239	0.318	0.774
454	Q	QEHRDGLY	0.489	0.248	0.356	0.804
492	R	RK	0.509	0.215	0.323	0.815
387	I	TVIL	0.515	-0.236	0.36	0.815
232	D	DNTHAEQS	0.531	-0.185	0.294	0.815
395	G	GSTANDQFHK	0.543	0.19	0.311	0.815
237	T	TKMES	0.58	-0.173	0.311	0.815
483	K	KR	0.602	-0.156	0.297	0.815
337	I	VIL	0.609	0.187	0.362	0.815
522	K	KREGMQ	0.609	0.15	0.292	0.815
399	F	FLISV	0.612	-0.154	0.301	0.815
576	D	NDSGEKTACPV	0.612	0.164	0.321	0.815
438	I	ILMV	0.631	-0.16	0.331	0.815
497	V	VI	0.636	-0.161	0.337	0.815
576b	R	RKSNGQDHTYEP	0.643	0.179	0.382	0.815
464	F	FSHAYN	0.673	0.12	0.283	0.815
578	D	DGHNRCPS	0.689	-0.137	0.34	0.815
641	T	TDSENA	0.694	-0.117	0.296	0.815
481	D	DENAGH	0.701	-0.118	0.306	0.815
574	G	GEDRAKPST	0.728	0.102	0.292	0.829
431	D	DEA	0.734	-0.1	0.293	0.829
405	P	PLQRVMAKSTW	0.754	0.094	0.297	0.835
528	T	TNSDGQ	0.775	-0.096	0.333	0.841
202	V	VI	0.782	-0.082	0.294	0.841
575	G	GEKRMQS	0.791	0.088	0.33	0.841
223	T	TAI	0.869	-0.056	0.34	0.851

The tested site	The most associated amino acid	Amino acids in reducing frequency order at the tested site	P value	Effect size	Standard error	q value
580	F	FLIHVAMSTY	0.875	-0.048	0.304	0.851
591	E	EDGAK	0.893	-0.045	0.335	0.851
391	A	SATNLQR	0.894	0.049	0.365	0.851
442	F	FIVL	0.908	-0.04	0.349	0.857
208	S	SP	0.98	-0.009	0.356	0.876

Table S4. Statistical analysis of the association between E1/E2 amino acid variations and antibody neutralization.

The table presents results from linear regression models testing the association between antibody binding and polymorphisms at 77 sites (in total 123 residues were tested at these 77 sites, but only the most associated residue per site is shown in the table, numbered according to the H77 reference sequence). P values, effect sizes, and standard errors are reported, along with q-values representing FDR-corrected p-values to account for multiple testing. Associations at 20% FDR are highlighted in bold.

Amino acid at site 653	Count	Percent
D	406	80.08%
N	90	17.75%
E	10	1.97%

Amino acid at site 501	Count	Percent
S	181	35.70%
N	159	31.36%
T	59	11.64%
D	30	5.92%
G	19	3.75%
K	19	3.75%
E	17	3.35%
R	16	3.16%
Q	4	0.79%
H	1	0.20%

Amino acid at site 533	Count	Percent
E	402	79.29%
K	52	10.26%
A	14	2.76%
Q	13	2.56%
D	11	2.17%
V	8	1.58%
T	2	0.39%
N	1	0.20%
P	1	0.20%

Table S5. The frequency of different amino acids at each site (site 653, 501, 533) using 507 gt3a isolates in the BOSON cohort.
The top, medium and bottom tables indicate the frequency of different amino acids at site 653, site 501 and at site 533 respectively in the BOSON dataset. The counts and percent for each amino acid in 507 gt3a samples from BOSON cohort are shown in the tables.

Total number of potential glycosylation sites	Patients
13	2
14	1
15	48
16	3

Table S6. The total count of potential N-linked glycosylation sites detected in each patient. Most patients (48) exhibit 15 potential glycosylation sites, followed by a smaller number with 16, 13, or 14 sites.

Binding/ Neutralization	N-linked glycosylation sites	Most associated Motif	p value	Effect size	Standard error	q value
**binding**	**N476**	**NIT**	**0.042**	**0.564**	**0.270**	**0.170**
binding	N234	NTS	0.095	-0.430	0.252	0.228
binding	N532	NET	0.243	0.307	0.259	0.407
binding	N576c	NGS	0.280	-0.326	0.298	0.407
binding	N423	NRT	0.959	-0.014	0.272	0.991
binding	N430	NES	0.991	0.003	0.268	0.991
**neutralization**	**N476**	**NIT**	**0.011**	**0.775**	**0.291**	**0.094**
**neutralization**	**N234**	**NTS**	**0.016**	**-0.677**	**0.269**	**0.094**
**neutralization**	**N532**	**NET**	**0.058**	**0.545**	**0.280**	**0.173**
neutralization	N576c	NGS	0.220	0.409	0.328	0.407
neutralization	N423	NRT	0.306	-0.308	0.297	0.407
neutralization	N430	NES	0.568	-0.162	0.282	0.682

Table S7. Association between the tested glycosylation motifs at 6 glycosylation sites and antibody response.

The table indicates the *p* values, effect size, standard error from the linear regression for each test of the association between the absence or presence of E1/E2 N-linked glycosylation motifs and antibody binding and neutralization response. The location of the tested sites is relative to H77 polyprotein numbering. Associations at 20% FDR are highlighted in bold.
